# Comparisons of benefits and risks of single embryo transfer versus double embryo transfer: a systematic review and meta-analysis

**DOI:** 10.1186/s12958-022-00899-1

**Published:** 2022-01-27

**Authors:** Shujuan Ma, Yangqin Peng, Liang Hu, Xiaojuan Wang, Yiquan Xiong, Yi Tang, Jing Tan, Fei Gong

**Affiliations:** 1 Clinical Research Center for Reproduction and Genetics in Hunan Province, Reproductive and Genetic Hospital of CITIC-Xiangya, No. 567, Tongzipo West Road, Yuelu District, Changsha, 410205 China; 2 Chinese Evidence-based Medicine Center, West China Hospital, Sichuan University, No. 37, Guoxue Lane, Wuhou District, Chengdu, 610041 China

**Keywords:** Single embryo transfer, Double embryo transfer, Live birth rate, Multiple pregnancy rate, Perinatal complication

## Abstract

**Background:**

Evidence referring to the trade-offs between the benefits and risks of single embryo transfer (SET) versus double embryo transfer (DET) following assisted reproduction technology are insufficient, especially for those women with a defined embryo quality or advanced age.

**Methods:**

A systematic review and meta-analysis was conducted according to PRISMA guidelines. PubMed, EMBASE, Cochrane Library and ClinicalTrials.gov were searched based on established search strategy from inception through February 2021. Pre-specified primary outcomes were live birth rate (LBR) and multiple pregnancy rate (MPR). Odds ratio (OR) with 95% confidence interval (CI) were pooled by a random-effects model using R version 4.1.0.

**Results:**

Eighty-five studies (14 randomized controlled trials and 71 observational studies) were eligible. Compared with DET, SET decreased the probability of a live birth (OR = 0.78, 95% CI: 0.71–0.85, *P <* 0.001, *n* = 62), and lowered the rate of multiple pregnancy (0.05, 0.04–0.06, *P <* 0.001, *n* = 45). In the sub-analyses of age stratification, both the differences of LBR (0.87, 0.54–1.40, *P* = 0.565, *n* = 4) and MPR (0.34, 0.06–2.03, *P* = 0.236, *n* = 3) between SET and DET groups became insignificant in patients aged ≥40 years. No significant difference in LBR for single GQE versus two embryos of mixed quality [GQE + PQE (non-good quality embryo)] (0.99, 0.77–1.27, *P =* 0.915, *n* = 8), nor any difference of MPR in single PQE versus two PQEs (0.23, 0.04–1.49, *P =* 0.123, *n* = 6). Moreover, women who conceived through SET were associated with lower risks of poor outcomes, including cesarean section (0.64, 0.43-0.94), antepartum haemorrhage (0.35, 0.15-0.82), preterm birth (0.25, 0.21-0.30), low birth weight (0.20, 0.16-0.25), Apgar1 < 7 rate (0.12, 0.02-0.93) or neonatal intensive care unit admission (0.30, 0.14-0.66) than those following DET.

**Conclusions:**

In women aged < 40 years or if any GQE is available, SET should be incorporated into clinical practice. While in the absence of GQEs, DET may be preferable. However, for elderly women aged ≥40 years, current evidence is not enough to recommend an appropriate number of embryo transfer. The findings need to be further confirmed.

**Supplementary Information:**

The online version contains supplementary material available at 10.1186/s12958-022-00899-1.

## Background

Increasing success following assisted reproduction technology (ART) has been accompanied by concerns about high rates of multiple pregnancies [1]. Multiple pregnancy increased the risks of obstetric and neonatal complications, including but not limited to miscarriage, pre-eclampsia (PE), prematurity, low birth weight and perinatal mortality [2, 3]. Single embryo transfer (SET) is recommended to reduce the complications of multiple pregnancies following ART [4]. The American Society for Reproductive Medicine (ASRM) data from 2000 to 2017 shows that the proportion of SET has increased from 5.7 to 64.2%, among ART-conceived infants, meanwhile the percentage of multiple births has decreased from 53.1 to 26.4%, and simultaneous steady decreases in preterm birth and low birth weight rates have also been observed [5, 6]. However, this potential gain needs to be balanced against the risk of jeopardising the overall live birth rate (LBR). The latest Cochrane meta-analysis based on 12 randomized controlled trials (RCTs) shows that the chance of live birth was reduced in women undergoing SET compared with double embryo transfer (DET), the summarized relative risk (RR) was 0.67 (95% CI, 0.59–0.75) [7]. In addition, a large ART cycle dataset indicated that SET for any embryo transfer would result in a one-third lower LBR relative to DET [8]. Beyond these comparisons of overall LBR and multiple pregnancy rate (MPR), the studies did not further refine the population applicability of SET and DET, nor did they comprehensively assess the possible complications. Evidence referring to the trade-offs between live birth and multiple pregnancy following ART are insufficient.

It is well known that many patients idealize that twins would be their optimum outcome following ART [9], therefore DET is likely to remain part of clinical practice for the foreseeable future. The current issue arising is that for which patient cohorts are SET or DET most suited [10]. Due to the homogenous population and limited sample size, existing RCTs [11–24] and aggregated meta-analyses [4, 7] comparing the number of embryos transferred do not answer the above question. Clinical practice faces challenges of different patient age, cycles, embryo stages, embryo quality ratings etc., and would benefit from direction regarding which strategy would be most beneficial to specific subgroups. Moreover, although the latest ASRM guidelines have recommended a limit to the number of embryos transferred for different age groups, the evidence for this recommendation has not been disclosed and published [25]. More supporting evidence is therefore needed to assist ART program clinicians and patients.

Large observational studies focusing on the comparisons of different policies regarding the number of embryos transferred are emerging and do provide valid information [26–28]. The diverse population and large sample size offer the possibility of comprehensive subgroup analyses, involving different ages, embryo stages and embryo quality stratification, etc. [26, 29–31]. Additionally, longer follow-up times ensure the observation of reproductive, obstetric and perinatal outcomes. Thus, by integrating the information extracting from RCTs and observational studies, we investigated the overall effectiveness (e.g., LBR and MPR) of SET versus DET, and also focused on (i) whether transferring one or two embryos would be more beneficial to specific subgroups, especially in the consideration of embryo quality and maternal age, and (ii) assessing perinatal and neonatal complications following SET/DET as comprehensively as possible.

## Methods

This systematic review adhered to the PRISMA guidelines [32], and was prospectively registered on PROSPERO (registration ID: CRD42021258452). Institutional review board approval was not required as it was a meta-analysis.

### Inclusion criteria

RCTs and observational studies comparing benefits and risks of SET versus DET in a single cycle, in infertile women using their own oocytes and embryos were deemed eligible for inclusion. To prevent any confusion between per person and per cycle, studies were excluded if it was not possible to clarify that each woman was included only once.

### Literature search

A systematic electronic literature search was performed in PubMed, Embase, Cochrane Library, and RCT registries including ClinicalTrials.gov and WHO International Clinical Trials Registry Platform, through to February 9, 2021. The bibliographies of relevant studies and reviews were scrutinized for any additional eligible studies not covered by the literature search. The literature search combined the terms and descriptors related to human embryo transplantation concerning literature published in English (Supplemental file for full literature search). Conference abstracts and comments were not considered.

### Outcome measures

All the available reproductive, obstetric and perinatal outcomes were measured (Supplemental file for the definition of outcome). Pre-specified primary outcomes were LBR and MPR. The secondary outcomes included clinical pregnancy rate (CPR), miscarriage, birth weight, delivery gestational age, preterm birth, low birth weight, perinatal mortality, birth defect, caesarean section, gestational diabetes (GDM), PE, antepartum haemorrhage (APH), Apgar 1 < 7, neonatal intensive care unit (NICU) admission.

### Study selection and data extraction

Citations were merged in Microsoft Access Database to facilitate management. Duplicates were removed, and two reviewers independently applied the inclusion criteria to all retrieved citations in an un-blinded standardized manner, screened by title, abstract and full text successively. Data on characteristics of study (first author, publication year, location, study design and study period), population (participants, age, major inclusion and exclusion criteria), cycle (type of cycle, first or not and embryo stage), comparison categories and clinical outcomes (sample size, numbers of events and total, mean, standard deviation, risk estimates, 95% CIs, adjusted factors and conclusions) were extracted onto a piloted structured form by two reviewers, independently. The most comprehensive report would be given precedence if there were multiple publications from the same study or data source, while the others might be used as supplementary information. When studies had multiple comparisons, only the information and data of interest were extracted. Any uncertainty or disagreements were resolved through discussion and, if necessary, a consensus could be reached with the help of senior authors.

### Quality assessment and statistical analysis

The quality of included RCTs was assessed using the Cochrane risk of bias tool [33]. The Newcastle-Ottawa quality assessment scale (NOS) was used to assess the quality of included observational studies [34]. Comparisons were conducted between SET and DET groups in a single cycle. Considering the inclusion of both RCT and observational studies, meta-analyses were conducted using a Mantel-Haenszel (MH) random-effects model. To assess the possible impact of characteristics of the patients and embryos, subgroup analyses were pre-specified to separate the distinct types of study design (RCT or observational studies), cycle (fresh or frozen), embryo stage (cleavage or blastocyst), embryo quality rating and maternal age. Embryos/blastocysts were classified as good quality (GQE) or non-good quality (PQE) according to the standards established in the respective studies, and the subgroup comparisons were set to transplant a single GQE versus two GQEs (G/GG), a single GQE versus two embryos of mixed quality (GQE + PQE) (G/GP), and a single PQE versus two PQEs (P/PP). Maternal age was divided into three segments, with cut-offs being 35 and 40 years (*<* 35, 35-40, ≥40 years). Moreover, sensitivity analyses pooled of adjusted ORs, limited to first cycle or elective SET (eSET) cycles were performed to assess the robustness of the findings. Pooled effect size was deemed statistically significant at *P <* 0.05. Most data were dichotomous, we used the numbers of events in the groups of each study to calculate the ORs with 95% CIs. For continuous parameters, all the units had been harmonized by data conversion prior to analysis, the weighted mean differences (WMD) with 95% CIs were pooled to determine the effect size [35]. Heterogeneity was quantified using the estimated *I*^*2*^ statistic [36]. Publication bias was assessed using Begg’s test for analyses enrolling more than 10 studies [37]. The leave-one-out method was used to evaluate whether any single study dominated the findings. All statistical analyses were performed using R version 4.1.0.

## Results

### Description of included studies

**Fig. 1:**
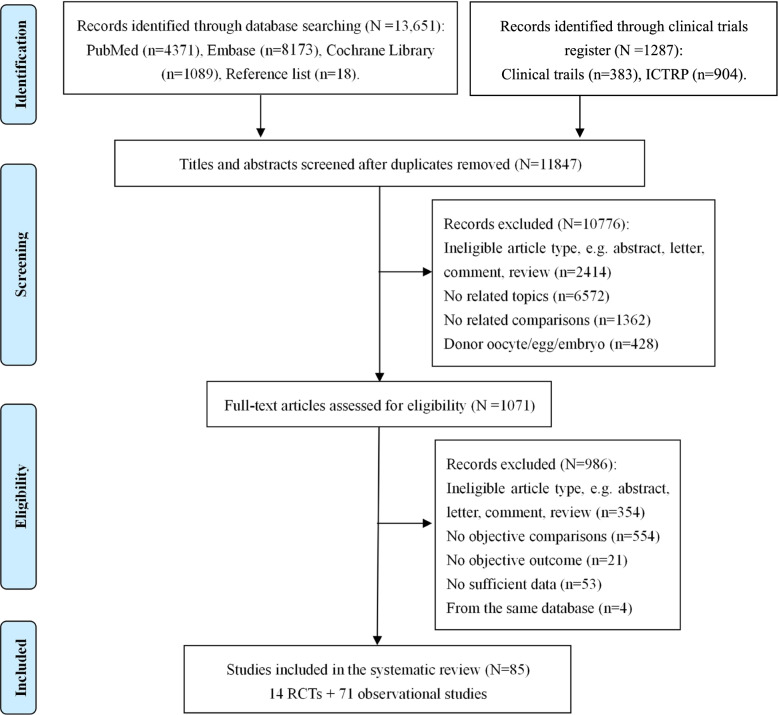
Flow diagram of study selection. DET, double embryo transfer; ICTRP, international Clinical Trials Registry Platform; SET, single embryo transfer; RCT, randomized controlled trial

The literature search retrieved 14,938 citations. After removing duplicates, 11,847 abstracts were reviewed and 1071 full-text articles were further assessed for eligibility. Finally, 85 articles [2, 11–24, 26–31, 38–101] involving 339,492 participants, provided extractable data for the quantitative meta-analysis (Fig. 1), including 14 RCTs and 71 observational studies; of which, data extracted by two RCTs [14, 20] came from four references [14, 20, 63, 92], and the other two RCT studies [16, 23] provided both the results of RCTs and observational cohorts. The characteristics of the included studies are presented in Supplementary Table S1. Women from 24 studies were recruited during their first cycle and 44 studies provided data on eSET. Most studies clarified the type of cycles (69 fresh, 11 frozen and 5 both) and the stage of embryo transfer (34 cleavage, 24 blastocyst, 23 both and 4 unclear). Thirteen studies provided results of stratified comparison based on embryo quality (12 G/GG, 11 G/GP and 8 P/PP), and 22 studies were included in the sub-analyses considering different age groups (17 aged < 35, 6 aged 35–40 and 5 aged ≥40 years). The results of quality assessment are presented in Supplementary Table S2. Twelve RCTs reported a randomization method, five carried out allocation concealment and six executed blinding for participants or personnel. Nine observational studies were awarded six stars in quality assessment, 47 studies were graded seven stars and 15 studies were marked eight stars.

### Primary outcomes

#### Live birth rate

Sixty-two studies [11–20, 22–24, 26–31, 38–41, 44, 45, 47–53, 56, 58, 60–62, 64–66, 69, 71, 73–75, 77–84, 86–91, 94, 99] demonstrated a reduced LBR after SET (28,529/85,988, 33.2%) than that after DET (113,658/247,116, 46.0%) in a single cycle (OR = 0.78, 95% CI: 0.71–0.85, *I*^*2*^ = 91%, *P <* 0.001) (Supplementary Fig. S1). The analysis results are presented in Table 1. No publication bias was detected by Begg’s test (*P* = 0.062).

**Table 1:** Analyses of primary outcomes between SET and DET in a single cycle

	Live birth rate						Multiple pregnancy rate					
	Studies no.	SET total	DET total	***I***^***2***^	OR (95%CI)	***P*** value	Studies no.	SET total	DET total	***I***^***2***^	OR (95%CI)	***P*** value
**Overall**	62	85,988	247,116	91%	0.78 (0.71-0.85)	< 0.001	45	17,979	49,645	2%	0.05 (0.04-0.06)	< 0.001
**Subgroup analyses**												
Maternal age (years)						0.504^*^						0.035^*^
< 35^a^	12	20,637	27,263	85%	0.71 (0.61-0.84)	< 0.001	11	5061	12,322	0%	0.03 (0.03-0.05)	< 0.001
35-40	6	11,009	20,285	69%	0.80 (0.69-0.94)	0.005	5	3767	15,100	0%	0.04 (0.03-0.06)	< 0.001
≥ 40	4	2584	3395	69%	0.87 (0.54-1.40)	0.565	3	347	528	0%	0.34 (0.06-2.03)	0.236
Quality						–						–
G/GG	7	7293	24,514	82%	0.63 (0.52-0.77)	< 0.001	10	4698	3665	57%	0.06 (0.03-0.10)	< 0.001
G/GP	8	7601	3308	81%	0.99 (0.77-1.27)	0.915	9	4648	1470	66%	0.12 (0.06-0.26)	< 0.001
P/PP	3	786	681	0%	0.57 (0.45-0.71)	< 0.001	6	381	429	54%	0.23 (0.04-1.49)	0.123
Embryo stage						0.116^*^						0.28^*^
Cleavage	32	40,348	140,827	96%	0.67 (0.55-0.82)	< 0.001	17	5681	32,187	0%	0.06 (0.04-0.08)	< 0.001
Blastocyst	25	39,455	93,424	88%	0.81 (0.71-0.92)	0.001	22	9308	14,928	25%	0.05 (0.04-0.06)	< 0.001
Cycle						0.608^*^						0.712^*^
Fresh	53	77,288	217,979	91%	0.80 (0.72-0.88)	< 0.001	36	14,391	45,104	0%	0.05 (0.05-0.07)	< 0.001
Frozen	10	8541	28,928	89%	0.74 (0.59-0.94)	0.013	7	3057	3885	48%	0.05 (0.03-0.09)	< 0.001
Design						< 0.001^*^						0.73^*^
RCT	13	1044	1050	0%	0.53 (0.44-0.63)	< 0.001	5	222	234	0%	0.06 (0.02-0.21)	< 0.001
Observational study	49	84,944	246,066	93%	0.82 (0.75-0.90)	< 0.001	40	17,757	49,411	11%	0.05 (0.04-0.06)	< 0.001
**Sensitivity analyses**												
Adjusted	12	6921	28,828	41%	0.77 (0.68-0.87)	< 0.001	4	797	1847	0%	0.06 (0.02-0.16)	< 0.001
First cycle	21	76,470	235,620	96%	0.70 (0.61-0.81)	< 0.001	10	11,000	41,814	54%	0.05 (0.03-0.07)	< 0.001
eSET	35	31,189	29,418	70%	0.80 (0.72-0.89)	< 0.001	27	4245	5522	0%	0.05 (0.03-0.07)	< 0.001

Note: ^a^included one study (Chai 2014) whose participants aged less than or equal to 35 years

Abbreviation: CI, confidence interval; DET, double embryo transfer; eSET, elective single embryo transfer; G/GG, a single good quality embryo (GQE) compared with two GQEs; G/GP, a single GQE compared with two embryos of mixed quality (GQE + PQE); OR, odds ratio; P/PP, a single non-top quality embryo (PQE) compared with two PQEs; RCT, randomized controlled trial; SET, single embryo transfer

*P for interaction

**Fig. 2:**
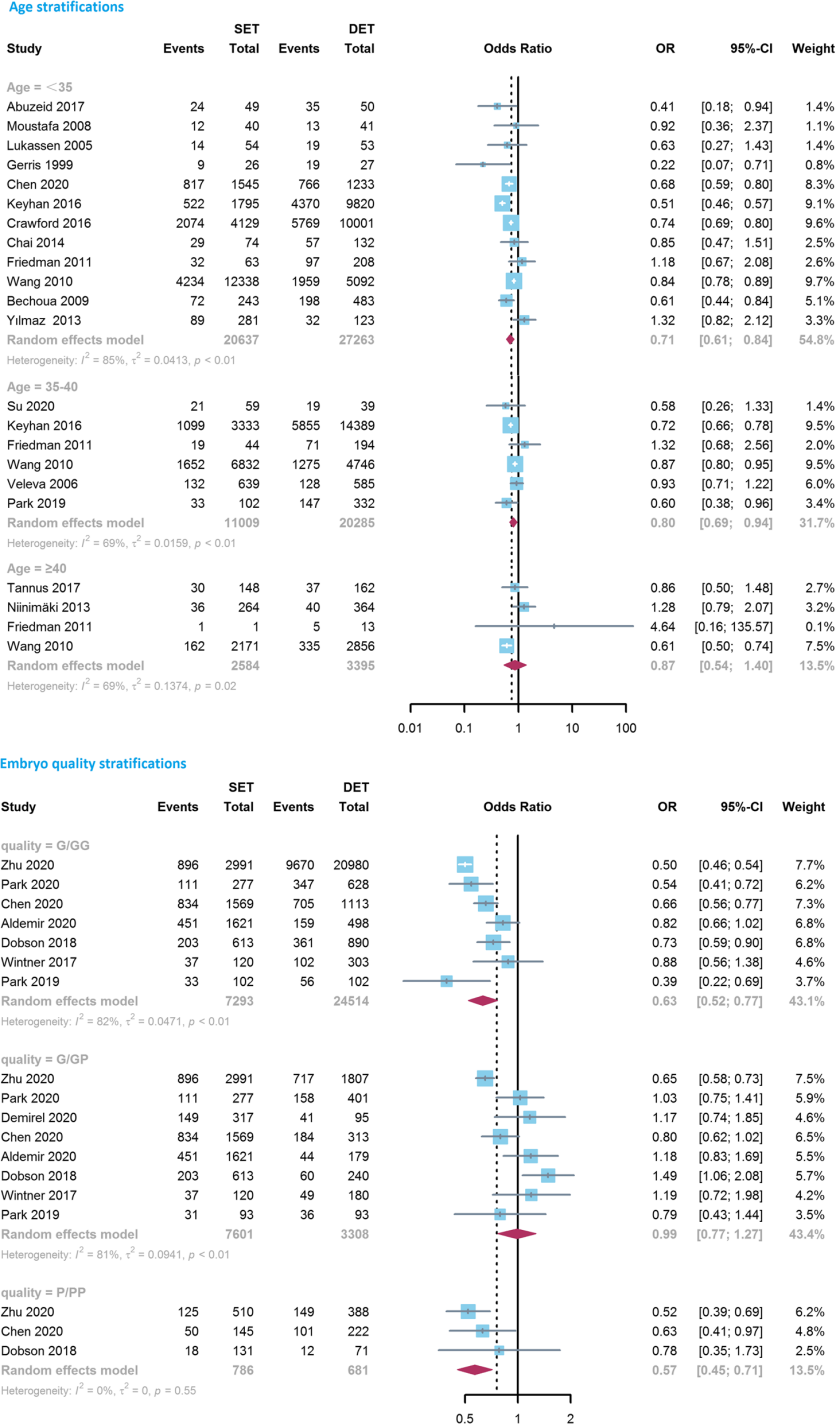
Forest-plot comparing the live birth rate between single embryo transfer (SET) and double embryo transfer (DET) based on maternal age and embryo quality stratification. G/GG, a single good quality embryo (GQE) versus two GQEs; P/PP, a single non-good quality embryo (PQE) versus two PQEs; G/GP, a single GQE versus two embryos of mixed quality (GQE + PQE)

Among subgroup analyses, when considering different age groups of included patients, an increase in LBR favoring the DET group was noted for patients aged < 35 years (0.71, 0.61–0.84, *I*^*2*^ = 85%, *n* = 12) and 35–40 years (0.80, 0.69–0.94, *I*^*2*^ = 69%, *n* = 6), but no difference was observed for patients ≥40 years old (0.87, 0.54–1.40, *I*^*2*^ = 69%, *P* = 0.565, *n* = 4; Fig. 2). The difference in the comparison of LBR between groups decreased with age stratification. When considering embryo quality, the LBR was significantly decreased after transferring a single good quality embryo (GQE) compared with two GQEs (0.63, 0.52–0.77, *I*^*2*^ = 82%, *n* = 7), as well as a single PQE compared with two PQEs (0.57, 0.45–0.71, *I*^*2*^ = 0%, *n* = 3; Fig. 2). Meanwhile, no difference was noted between a single GQE and two embryos of mixed quality (GQE + PQE) (0.99, 0.77–1.27, *I*^*2*^ = 81%, *P* = 0.915, *n* = 8; Fig. 2). Subgroup analyses performed according to embryo stage (cleavage, blastocyst) and cycle type (fresh, frozen) suggested that, the chance of live birth in the DET group was significantly greater than that in the SET group in all the subgroups, with ORs being 0.67 (*P* < 0.001, *n* = 32), 0.81 (*P* = 0.001, *n* = 25), 0.80 (*P <* 0.001, *n* = 53) and 0.74 (*P* = 0.013, *n* = 10), respectively. Sub-analysis concerning study design indicated that the combination of RCTs resulted in a lower OR (0.53, 0.44–0.63, *I*^*2*^ = 0%, *n* = 13), compared with the result obtained from observational studies (0.82, 0.75–0.90, *I*^*2*^ = 93%, *n* = 49, *P* for interaction *<* 0.001).

#### Multiple pregnancy rate

Forty-five studies [2, 11, 13, 15, 21, 24, 28–31, 38, 39, 43, 44, 48–50, 53, 54, 56–59, 62, 64, 66–69, 72, 75–77, 79–81, 85–87, 91, 93–96, 98] were pooled and found that the MPR was significantly lower in the SET group than in the DET group (0.05, 0.04–0.06, *I*^*2*^ = 2%, *P <* 0.001) (Supplementary Fig. S2). This suggested that for a woman with a 16.7% (8314/49,645) chance of multiple pregnancy following a single cycle of DET, the rate following a single SET would be between 0.7 and 1.0%. The analysis results are presented in Table 1. No publication bias was detected by Begg’s test (*P* = 0.531).

**Fig. 3:**
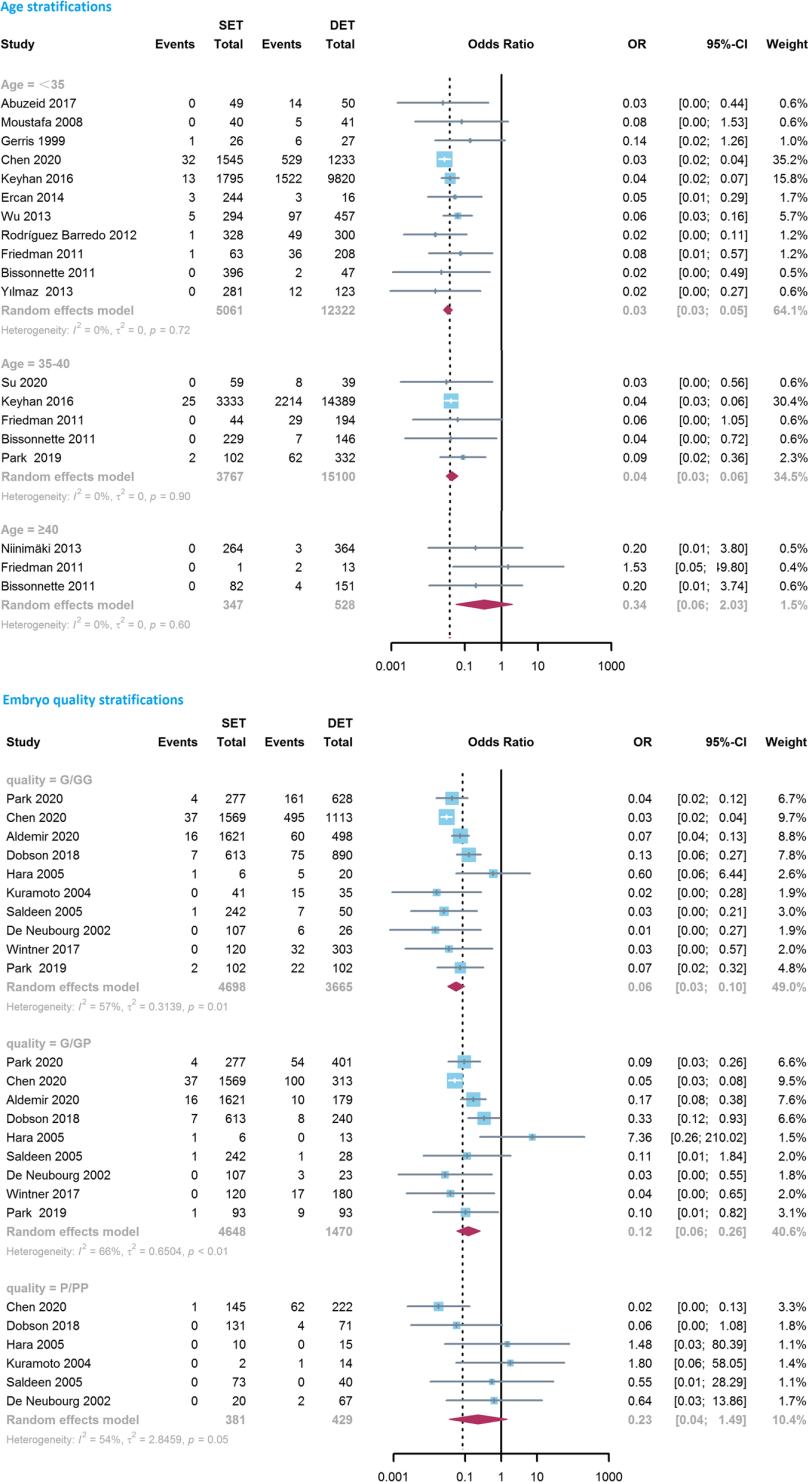
Forest-plot comparing the multiple pregnancy rate between single embryo transfer (SET) and double embryo transfer (DET) based on maternal age and embryo quality stratification. G/GG, a single good quality embryo (GQE) versus two GQEs; P/PP, a single non-good quality embryo (PQE) versus two PQEs; G/GP, a single GQE versus two embryos of mixed quality (GQE + PQE)

In the subgroup analyses of age stratification (Fig. 3), the differences of MPR between SET and DET were stable and significant in women aged < 35 (0.03, 0.03–0.05, *I*^*2*^ = 0%, *P <* 0.001, *n* = 11) and 35–40 years (0.04, 0.03–0.06, *I*^*2*^ = 0%, *P* < 0.001, *n* = 5), whereas the aggregate result from three studies found that the difference became insignificant in patients aged ≥40 years (0.34, 0.06–2.03, *I*^*2*^ = 0%, *P* = 0.236, *n* = 3). Similarly, 10 observational studies investigated the differences in the association between MPR and the number of transferred embryos by embryo quality grade (Fig. 3). In comparison with a single GQE, transferring two GQEs and two embryos of mixed quality (GQE + PQE) both led to significantly higher MPRs, the ORs were 0.06 (0.03–0.10, *I*^*2*^ = 57%, *P <* 0.001, *n* = 10) and 0.12 (0.06–0.26, *I*^*2*^ = 66%, *P* < 0.001, *n* = 9), respectively. However, the difference in MPR between a single PQE and two PQEs was reduced and was no longer be statistically significant (0.23, 0.04–1.49, *I*^*2*^ = 54%, *P* = 0.123, *n* = 6). Moreover, the pooled results did not materially change in the subgroup analyses regarding embryo stage (cleavage, blastocyst), cycle type (fresh, frozen) and study design (RCT, observational study), with pooled ORs being 0.05 (*P <* 0.001) or 0.06 (*P <* 0.001).

### Secondary Outcomes

Table 2 summarizes the overall analysis results of secondary maternal pregnancy outcomes and neonatal outcomes.

**Table 2:** Overall analyses of secondary outcomes between SET and DET in a single cycle

Index	Studies no.	SET total	DET total	***I***^***2***^	OR/WMD (95%CI)	***P*** value
**Maternal pregnancy outcomes and complications**						
CPR	60	43,414	68,289	82%	0.78 (0.71-0.85)	< 0.001
Miscarriage rate	43	5230	13,249	30%	1.10 (0.95-1.27)	0.211
Perinatal mortality rate	7	1135	1970	0%	0.71 (0.25-2.06)	0.532
Cesarean rate	3	238	698	15%	0.64 (0.43-0.94)	0.024
GDM rate	1	76	103	–	0.45 (0.02-11.11)	0.623
PE rate	1	76	103	–	9.86 (0.50-193.72)	0.132
APH rate	1	76	103	–	0.35 (0.15-0.82)	0.016
**Neonatal outcomes and complications**						
Delivery gestational age (week)	8	1136	1771	58%	0.88 (0.56-1.20)	< 0.001
Preterm birth rate^a^	13	1852	2380	0%	0.25 (0.21-0.30)	< 0.001
Birth weight (g)	7	1164	2572	84%	297.47 (208.47-386.46)	< 0.001
Low birth weight rate	9	1360	2826	0%	0.20 (0.16-0.25)	< 0.001
Birth defect rate	3	964	1672	0%	1.32 (0.68-2.59)	0.414
Apgar1 < 7 rate	2	99	193	0%	0.12 (0.02-0.93)	0.042
NICU admission rate	2	99	193	0%	0.30 (0.14-0.66)	0.003

Note: ^a^preterm birth rate was calculated as the number of preterm births divided by the total number of live births (multiple gestations included) in one of included studies (Sini 2020)

Abbreviation: *2SET* Two consecutive elective single embryo transfer; *APH* Antepartum haemorrhage; *CI* Confidence interval; *CPR* Clinical pregnancy rate; *DET* Double embryo transfer; *GDM* Gestational diabetes; *LBR* Live birth rate; *MBR* Multiple birth rate; *MPR* Multiple pregnancy rate; *NICU* Neonatal intensive care unit; *OR* Odds ratio; *PE* Pre-eclampsia; *WMD* Weighted mean difference

A significant decrease in CPR (37.4% vs. 48.0%, 0.78, 0.71–0.85, *I*^*2*^ = 82%, *P* < 0.001, *n* = 60) was noted in the SET group compared with the DET group [11–15, 19, 23, 24, 27–31, 38, 39, 41–44, 48–51, 53–59, 62, 64–67, 69, 70, 72, 74–81, 84, 86, 87, 90, 91, 93–98, 100, 101]. Several subgroup analyses indicated a different direction, no differences of CPR were noted between SET and DET in subgroups of patients aged ≥40 years (0.88, 0.59–1.30, *I*^*2*^ = 70%, *n* = 5), GQE versus GQE + PQE (1.01, 0.89–1.16, *I*^*2*^ = 0%, *n* = 9), and frozen cycles (0.79, 0.62–1.00, *I*^*2*^ = 83%, *n* = 9; Supplementary Table S3, Fig. S3). Forty-three studies [11–14, 16, 19, 20, 23, 28–31, 38–44, 48, 50, 51, 53, 55, 57, 58, 62, 64–66, 69, 72, 74, 77, 79–81, 90, 91, 94, 95, 98] evaluated the miscarriage rates, and no significant difference was found between groups during the overall analysis (15.6% vs. 14.2%, 1.10, 0.95–1.27, *I*^*2*^ = 30%, *P* = 0.211), neither in all the conducted subgroups (Supplementary Table S4). Seven studies [19, 30, 39, 58, 65, 81, 94] provided information on perinatal mortality, and no difference was observed (0.3% vs 0.7%, 0.71, 0.25–2.06, *I*^*2*^ = 0%, *P* = 0.532). Compared with DET, mothers with SET had a lower risk of cesarean section (0.64, 0.43–0.94, *I*^*2*^ = 15%, *P* = 0.024, *n* = 3). Only one included study [39] reported on GDM, PE and APH in the late pregnancy, there was no significant difference between SET and DET groups with respect to the risk of GDM (0.0% vs. 1.0%, *P* = 0.623) and PE (3.9% vs. 0.0%, *P* = 0.132), meanwhile, APH rate was significantly lower in the SET group (10.5% vs. 25.2%, 0.35, 0.15-0.82, *P* = 0.016).

Eight studies [11, 15, 29, 30, 44, 53, 65, 81], including 2907 live birth cycles, provided data on continuous gestational age at birth. A significantly longer gestational age at birth was found in the SET group compared with the DET group (WMD =0.88 weeks, 95% CI: 0.56–1.20, *I*^*2*^ = 58%, *P* < 0.001). Moreover, 13 studies [11, 19, 23, 29–31, 38, 39, 44, 53, 65, 77, 81] evaluated preterm birth, and a significantly reduced probability of preterm birth was observed in the SET group (9.9% vs. 31%, 0.25, 0.21–0.30, *I*^*2*^ = 0%, *P* < 0.001). The overall findings did not materially change in all the conducted subgroup analyses (Supplementary Table S5). Similarly, the overall birth weight of live births in the SET group was significantly higher than that in the DET group in a single cycle (WMD =297.47 g, 95% CI: 208.47–386.46, I^2^ = 84%, *P* < 0.001, *n* = 7). The pooled analysis of low birth weight rate generated a total of 3962 live births from nine studies [19, 23, 29, 30, 38, 39, 44, 77, 81], and revealed a significant decrease in risk of low birth weight in the SET group (7.6% vs. 28.9%, 0.20, 0.16–0.25, I^2^ = 0%, *P* < 0.001), the finding was further confirmed by all the conducted subgroup analyses (Supplementary Table S6). Aggregated data from three studies [30, 44, 53] showed that there was no statistical difference in the risk of birth defects between groups (1.6% vs. 1.3%, *P* = 0.414), while two other studies [39, 81] reported statistically different risks of Apgar 1 < 7 rate (0.0% vs. 7.8%, 0.12, 0.02–0.93, *I*^*2*^ = 0%, *P* = 0.042, *n* = 2) and NICU admission rate (8.1% vs. 23.8%, 0.30, 0.14–0.66, *I*^*2*^ = 0%, *P* = 0.003, *n* = 2).

### Sensitivity Analyses

For the comparison of LBR, sensitivity analyses pooled of adjusted ORs (0.77, 0.68–0.87, *P <* 0.001, *n* = 12), limited to first cycle (0.70, 0.61–0.81, *P <* 0.001, *n* = 21) and eSET cycles (0.80, 0.72–0.89, *P <* 0.001, *n* = 35) confirmed the stability of the overall result (Table 1). The compared results of MPR were robust in all the pre-specified sensitivity analyses, with pooled ORs being 0.05 or 0.06 (*P <* 0.001; Table 1). No difference of CPR was noted between SET and DET in the summary of adjusted ORs (0.85, 0.61–1.18, *P* = 0.326, *n* = 6), while the sensitivity analyses limited to first cycle (0.68, 0.57–0.80, *P <* 0.001, *n* = 14) and eSET cycles (0.82, 0.73–0.92, *P* = 0.001, *n* = 35) confirmed the stability of the overall result (Supplementary Table S3). Similarly, except that a significantly higher risk of miscarriage was found in the SET group when the included studies were restricted to the first cycle (1.43, 1.10–1.86, *P* = 0.009, *n* = 10), no differences were found in all the other sensitivity analyses (Supplementary Table S4). Additionally, both the compared results of preterm birth rate and low birth weight rate were robust in all the pre-specified sensitivity analyses (Supplementary Table S5, 6).

## Discussion

This systematic review is the most complete assessment of the short-term and long-term outcomes of SET versus DET to date. The findings of overall effectiveness (e.g., LBR and MPR) of SET versus DET were consistent with current evidence. SET yielded less probability of a live birth (0.78, 0.71-0.85) than DET in a single cycle, while simultaneously reducing the rate of multiple gestation (0.05, 0.04-0.06). By contrast, the present study provided more comprehensive subgroup/sensitivity analyses and tracked more adverse fertility outcomes, further promoted the generation of individual program. Interestingly, changes emerged during several subgroup comparisons.

An important factor that needs to be considered during embryo transfer is maternal age, particularly given the age-dependent decrements in ovarian function [102]. Recruitment data indicated that the effect sizes of LBR and CPR between SET and DET groups gradually increased with increasing age. The increased benefit of a live birth, as well as a decreased rate of multiple pregnancy, favoring the DET group, were noted for patients aged < 35 years (0.71, 0.61–0.84; 0.03, 0.03–0.05) and 35–40 years (0.80, 0.69–0.94; 0.04, 0.03–0.06). At age ≥ 40 years, the differences in LBR (0.87, 0.54–1.40) and MPR (0.34, 0.06–2.03) between the groups were no longer statistically significant. The findings suggested that women < 40 years, including those aged 35–40 years, may have a lower possibility of multiple pregnancy after choosing SET. However, for elderly women ≥40 years old, current evidence is insufficient to recommend an appropriate number of embryos to be transferred. Knowing that older women would suffer greater rates of oocyte aneuploidy and a decline in uterine receptivity [8], women of advanced age are most likely to have multiple embryos transferred as recommended by the ASRM guidelines [25]. There are discrepancies between the current results and the recommendations of the guidelines [25] in terms of the recommended number of embryos to be transferred in women ≥35 years. There is still an argument to be made in favor of SET or DET in these certain situations, the choice must be jointly made by patients and physicians based on the patients’ desires, the individual’s chances of a twin pregnancy and success [103]. More researches need to be devoted to the research in advanced-age infertile women, taking into account the impact of age on reproductive function and that advancing age also leads to a greater risk of adverse pregnancy outcomes [104].

Regarding embryo quality, compared with two GQEs, transferring a single GQE gained a significantly lower possibility of multiple pregnancy (0.06, 0.03–0.10), as well as a lower rate of live birth (0.63, 0.52–0.77). Since the chance of a live birth after a single PQE was only about 57% of that achieved by two PQEs, and there was no statistical difference in the MPR between groups, choosing to the DET might be beneficial in the absence of GQEs. The comparison between a single GQE and two embryos of GQE + PQE indicated that the PQE along with GQE did not improve the effectiveness of transfer, but significantly increased the risks of perinatal complications, including MPR, preterm birth rate and low birth weight rate. The interaction was proposed to explain that pre-implantation embryos could affect the development of surrounding embryos through the release of specific growth factors [105, 106]. Moreover, our sensitivity analyses showed that, despite the same effect size of MPR, eSET produced a slightly reduced difference in LBR between groups, and the ratio was 80% of the DET group, this is perhaps an acceptable LBR on the premise that the MPR could be dramatically reduced. Thus, eSET/SET is recommended when there is one or more GQE, this is consistent with the ASRM guidelines, which recommend SET in cases where a euploid embryo is available [25].

With the apparent shift in the goal of ART, favorable perinatal outcomes have been regarded as crucial as a successful clinical pregnancy [39]. Compared with previously published meta-analyses [4, 7], in addition to the benefits, this study tracked more perinatal and neonatal complications following SET/DET. The findings showed that both the mothers and infants conceived through SET acquired reduced risks of poor outcomes, verified through the lower rates of cesarean section (0.64, 0.43-0.94), APH (0.35, 0.15-0.82), preterm birth (0.25, 0.21-0.30), low birth weight (0.20, 0.16-0.25), Apgar1 < 7 rate (0.12, 0.02-0.93) and NICU admission (0.30, 0.14-0.66). These were justified by similar findings of other studies [107–109]. As multiple pregnancy has been greatly associated with the above complications, the reduction of its incidence in ART cycles using SET might have contributed to the favorable perinatal and neonatal outcomes. In addition to the negative influences on perinatal outcomes, multiple pregnancy could also adversely impact the infant’s first year of life and potentially increase healthcare needs and cost of living throughout the neonatal period up to the age of 1 year [110]. These longer-term outcomes still need to be further tracked and studied, to provide a more complete evaluation and recommendations.

The strength of this study is the big data from observational researches enabled us to address concerns about the generalizability of data from RCTs to routine clinical care [111]. Additionally, we required that each woman was included only once in each included study, as the inclusion of all ART data with multiple cycles would have introduced statistical complexity as well as concern for potential bias [112]. However, several limitations of this study need to be addressed and merit further discussion. First, although multiple sensitivity and subgroup analyses were carried out, significant heterogeneity existed in some analyses. Second, many observational studies do not report the relative effects of adjustment, so we would only use unadjusted data for the main analyses, however, when we used the adjusted outcomes as sensitivity analyses, most of the results were consistent with the main analyses. Nevertheless, our aggregate data lacked information available to adjust some important confounding factors (such as reason for infertility, medical conditions etc.). Third, the problem of insufficient sample size existed in some of our results, such as the comparisons of GDM rate, PE rate, APH rate and NICU admission rate etc., the relatively small sample size may have limited the power to identify a real difference, which therefore requires cautious interpretation.

## Conclusions

In conclusion, based on the current evidence, SET yielded less probability of a live birth than DET in a single cycle, while simultaneously reducing the possibility of multiple gestation, as well as its related perinatal and neonatal complications. Subgroup analyses suggested that in women < 40 years old or any GQE is available, SET should be incorporated into clinical practice owing to the significant reduction in MPR and the acceptable LBR. Meanwhile, in the absence of GQEs, DET may be a preferable option because of the significant benefit of a live birth and the insignificant chance of multiple pregnancy. However, for elderly women ≥40 years old, current evidence is insufficient to recommend an appropriate number of embryos to transfer. Further high-quality RCTs or national registry-based cohort studies are still required to confirm these findings that will allow individualized transplantation strategies for different cohorts of infertile women.

## Supplementary Information

**Additional file 1.**

**Additional file 2: Supplementary Table S1.** The characteristics of the included studies. **Supplementary Table S2.** Methodological quality of included studies. **Supplementary Table S3.** Sensitivity and subgroup analyses comparing clinical pregnancy rate after SET and DET in a single cycle. **Supplementary Table S4.** Sensitivity and subgroup analyses comparing miscarriage rate after SET and DET in a single cycle. **Supplementary Table S5.** Sensitivity and subgroup analyses comparing preterm birth rate after SET and DET in a single cycle. **Supplementary Table S6.** Sensitivity and subgroup analyses comparing low birth weight rate after SET and DET in a single cycle.

**Additional file 3: Figure S1.** Forest-plot comparing live birth rate after single embryo transfer (SET) and double embryo transfer (DET) in a single cycle.

**Additional file 4: Figure S2.** Forest-plot comparing multiple pregnancy rate after single embryo transfer (SET) and double embryo transfer (DET) in a single cycle.

**Additional file 5: Figure S3.** Forest-plot comparing the clinical pregnancy rate between single embryo transfer (SET) and double embryo transfer (DET) based on maternal age and embryo quality stratification. G/GG, a single good quality embryo (GQE) versus two GQEs; P/PP, a single non-good quality embryo (PQE) versus two PQEs; G/GP, a single GQE versus two embryos of mixed quality (GQE + PQE).

## Data Availability

All data generated or analyzed during this study are included in this published article and its supplementary information files.

## Abbreviations

ART: Assisted reproduction technology
APH: Antepartum haemorrhage
ASRM: The American Society for Reproductive Medicine
CI: Confidence interval
CPR: Clinical pregnancy rate
DET: Double embryo transfer
GDM: Gestational diabetes
GQE: Good quality embryos/blastocysts
LBR: Live birth rate
MPR: Multiple pregnancy rate
NICU: Neonatal intensive care unit
NOS: The Newcastle-Ottawa quality assessment scale
OR: Odds ratio
PE: Pre-eclampsia
PQE: Non-good quality embryos/blastocysts
RCT: Randomized controlled trial
RR: Relative risk
SET: Single embryo transfer
WMD: Weighted mean difference
