# Supplementary material for: Comparisons of benefits and risks of single embryo transfer versus double embryo transfer: a systematic review and meta-analysis
Source: Reprod Biol Endocrinol. 2022 Jan 27;20:20. doi: 10.1186/s12958-022-00899-1 (PMC8793185; doi:10.1186/s12958-022-00899-1)
Supplement: Supplementary file 1 — Additional file 1. [file 12958_2022_899_MOESM1_ESM.docx]

**[Search](D:/info/YoudaoDict_setup_3987/%E6%9C%89%E9%81%93%E8%AF%8D%E5%85%B8%E5%AE%89%E8%A3%85%E6%96%87%E4%BB%B6/Dict/7.5.1.0/resultui/dict/?keyword=search)**[**strategy**](D:/info/YoudaoDict_setup_3987/%E6%9C%89%E9%81%93%E8%AF%8D%E5%85%B8%E5%AE%89%E8%A3%85%E6%96%87%E4%BB%B6/Dict/7.5.1.0/resultui/dict/?keyword=strategy)

(((((((((((((Assisted Reproductive Techn*) OR (assisted conception)) OR (In vitro fertilization*)) OR (In-vitro fertilization*)) OR (Fertilization in Vitro)) OR (IVF)) OR (Intracytoplasmic Sperm Injection)) OR (Intracytoplasmic Sperm transfer)) OR (ICSI)) OR (embryo trans*)) OR (blastocyst trans*)) OR (embryo replace*)) OR (blastocyst replace*)) AND ((((((((((((((((((((((((((((("Single embryo") OR ("single blastocyst")) OR ("one embryo")) OR ("one blastocyst")) OR (Single-embryo)) OR (single-blastocyst)) OR (one-embryo)) OR (one-blastocyst)) OR (SET)) OR (SBT)) OR (eSET)) OR (eSBT)) OR ("double embryo*")) OR ("double blastocyst*")) OR ("2 embryo*")) OR ("2 blastocyst*")) OR ("two embryo*")) OR ("two blastocyst*")) OR (double-embryo*)) OR (double-blastocyst*)) OR (2-embryo*)) OR (2-blastocyst*)) OR (two-embryo*)) OR (two-blastocyst*)) OR (DET)) OR (DBT)) OR (eDET)) OR (eDBT)))

**Definition of outcome**

Live birth rate: LBR, defined as the number of deliveries that resulted in at least one live born baby per cycle;

Multiple pregnancy rate: MPR, defined as a clinical pregnancy with more than one intrauterine fetus per transfer cycle;

Clinical pregnancy rate: CPR, defined as pregnancy diagnosed by ultrasonographic intrauterine visualization or definitive clinical signs of pregnancy per transfer cycle;

Miscarriage rate: Defined as the spontaneous loss of a clinical pregnancy that occurs before 20 completed weeks of gestation;

Preterm birth rate: Defined as a birth that takes place after 22 but before 37 completed weeks of gestational age per live birth cycle;

Low birth weight rate: Defined as number of babies with birth weight <2500 g divided by the total number of live birth babies;

Perinatal mortality rate: Defined as the number of perinatal deaths divided the total number of fetuses, including stillbirths and live births.
