# Supplementary material for: Comparisons of benefits and risks of single embryo transfer versus double embryo transfer: a systematic review and meta-analysis
Source: Reprod Biol Endocrinol. 2022 Jan 27;20:20. doi: 10.1186/s12958-022-00899-1 (PMC8793185; doi:10.1186/s12958-022-00899-1)
Supplement: Supplementary file 2 — Additional file 2: Supplementary Table S1. The characteristics of the included studies. Supplementary Table S2. Methodological quality of included studies. Supplementary Table S3. Sensitivity and subgroup analyses comparing clinical pregnancy rate after SET and DET in a single cycle. Supplementary Table S4. Sensitivity and subgroup analyses comparing miscarriage rate after SET and DET in a single cycle. Supplementary Table S5. Sensitivity and subgroup analyses comparing preterm birth rate after SET and DET in a single cycle. Supplementary Table S6. Sensitivity and subgroup analyses comparing low birth weight rate after SET and DET in a single cycle. [file 12958_2022_899_MOESM2_ESM.docx]

**Supplementary Tables**

Supplementary Table S1……………………………………………………………………………1

Supplementary Table S2………………………………….……………………………………….24

Supplementary Table S3…………………………………………………………………………..27

Supplementary Table S4…………………………………………………………………………..28

Supplementary Table S5…………………………………………………………………………..29

Supplementary Table S6…………………………………………………………………………..30

| **Supplementary Table S1. The characteristics of the included studies.** | | | | | | | | | | | |
| --- | --- | --- | --- | --- | --- | --- | --- | --- | --- | --- | --- |
| **Study (year)** | **Design** | **Location** | **Study period** | **Participants** | **Cycle** | **First cycle** | **Embryo stage** | **Comparison** | **Total number** | **Age (mean/ median/ range)** | **Clinical outcomes** |
| Abuzeid 2017 | RCT | USA | 2009.01 - 2013.10 | **Included:** <35 years, day 3 FSH <10 mIU/ml, no history of POR, ≤1 previous IVF failure, no uterine cavity abnormalities and no contraindication to treatment medications or procedures; **Excluded:** had uterine factors such as submucous fibroid, endometrial polyps, uterine septum or significant arcuate uterine anomaly. | Fresh | No | Blastocyst | eSBT | 49 | 29.4 ± 3.3 | CPR, MPR, LBR, MBR, implantation rate, miscarriage rate, ectopic pregnancy rate, preterm birth rate, early and extremely preterm birth rate, gestational days at birth. |
|  |  |  |  |  |  |  |  | DBT | 50 | 30.6 ± 2.8 |  |
| Prados 2015 | Open-label RCT | Spain | 2008.12 - 2009.09 | **Included:** <38 years, first trial of IVF or ICSI; had ≥4 good-quality embryos on D3; **Excluded:** donation treatments, PGD, no apermatozoa obtained. | Fresh | Yes | Cleavage/Blastocyst | eSET on D3 | 50 | 33.0 ± 2.9 | CPR, LBR, MBR, implantation rate, miscarriage rate, live born rate per embryo. |
|  |  |  |  |  |  |  |  | eSET on D5 | 50 | 33.6 ± 2.6 |  |
|  |  |  |  |  |  |  |  | eDET on D3 | 49 | 33.0 ± 2.4 |  |
|  |  |  |  |  |  |  |  | eDET on D5 | 50 | 32.7 ± 3.3 |  |
| López-Regalado 2014 (a) | RCT | Spain | 2010.01 - 2012.12 | **Included:** <38 years, BMI 19-29 kg/m^2^, D3 FSH<15mUI/ml, first cycle of IVF/ICSI or second cycle after a prior attempt with a positive pregnancy test result; **Excluded:** ≥ 5 years of infertility, previous surgery, uterine malformations, ≥2 repeated spontaneous abortions. | Fresh/Frozen | First/ Second | Cleavage | eSET | 84 | 32.2 ± 3.6 (21-37) | CPR, MPR, LBR, MBR, abortion rate weeks of gestation at delivery. |
|  |  |  |  |  |  |  |  | DET | 91 | 31.7 ± 3.8 (20-37) |  |
| Forman 2013 | Non-inferiority RCT | USA | 2011.08 - 2012.06 | **Included:** ≤ 42 years, ≤1 prior failed IVF cycle, a normal endometrial cavity, normal ovarian reserve, ≥2 expanded blastocysts; **Excluded:** severe male factor infertility requiring surgical sperm extraction, anovulatory PCOS, BMI > 30 kg/m^2^. | Fresh/Frozen | No | Blastocyst | eSBT (Euploid) | 89 | 35.1 ± 3.9 (25.1-41.4) | CPR, LBR, single birth rate, MBR, clinical miscarriage rate. |
|  |  |  |  |  |  |  |  | DBT (untested) | 86 | 34.5 ± 4.7 (22.9-42.6) |  |
| Moustafa 2008 | Open-label RCT | Egypt | 2004.09-2006.09 | **Included:** fresh cycle, ≥1 good quality embryo (Grade I–II), ≤ 30 years, no contraindication for pregnancy;  **Excluded:** only poor quality embryos available for transfer, refusal to consent or participate in the clinical trial. | Fresh | NA | Cleavage | eSET | 40 | 25.1 ± 3.0 (20-30) | CPR, MPR, LBR, gestational days at birth. |
|  |  |  |  |  |  |  |  | DET | 41 | 25.4 ± 3.2 (20-30) |  |
| van Montfoort 2006 | Patient-blind RCT | The Netherlands | 2002.01-2004.12 | **Included:** first treatment cycle, ≥2 normally fertilized oocytes;  **Excluded:** PGD, eSET for medical reasons, could not be informed adequately because of a language barrier. | Fresh | Yes | Cleavage | eSET | 154 | 32.7 ± 3.3 | Positive HCG rate, abortion <13 weeks rate, ongoing CPR, LBR, MBR. |
|  |  |  |  |  |  |  |  | DET | 154 | 32.4 ± 3.3 |  |
| Bhattacharya 2006  (ECOSSE) | Multi-center  RCT | UK | 2005.01-2005.11 | **Included:** ≤37years, first or second IVF/ICSI cycle, ≥4 good quality embryos at time of embryo transfer; **Exclusion :** women undergoing PGD, or assisted hatching, or a history of ≥3 recurrent miscarriage, or multiple IVF failure, or donor or recipient of gametes, or previous non-IVF live birth. | Fresh | First/ Second | Cleavage | eSET | 11 | ≤37 | LBR, MBR. |
|  |  |  |  |  |  |  |  | DET | 12 | ≤37 |  |
| Thurin 2005 | Multi-center double-blind RCT | Sweden | NA | **Included:** ≥36 years. First or second IVF/ICSI cycle. ≥2 good quality embryos available | Fresh | First/ Second | Cleavage + blastocyst | eSET | 20 | ≥36 | LBR, MBR, cLBR. |
|  |  |  |  |  |  |  |  | DET | 22 | ≥36 |  |
| Lukassen 2005 | Open-label RCT | The Netherlands | 2001.01-2003.02 | **Included:** ﬁrst IVF/ICSI cycle ever or the first cycle after a successful treatment, < 35 years, basal FSH level < 10 IU/l, ≥2 good embryos available for transfer on D3; **Excluded:** with a medical reason for elective SET. | Fresh | First/ Second | Cleavage | SET | 54 | 30.2 ± 3.2 (20-34) | CPR, miscarriage rate, ectopic pregnancy rate, LBR, MBR, perinatal death rate, preterm birth rate, low birthweight rate. |
|  |  |  |  |  |  |  |  | DET | 53 | 31.2 ± 2.9 (25-34) |  |
| Thurin 2004 / Kjellberg 2006 | Multi-center, double-blind RCT | Sweden | 2000.05-2003.10 | **Included:** < 36 years at the time of the transfer of fresh embryo, first or second IVF cycle, had ≥2 embryos of good quality available. | Fresh/Frozen | First/ Second | Cleavage/Blastocyst | eSET | 330 | 30.9 ± 3.0 (22.6–35.9) | Ectopic pregnancy rate, miscarriage rate, ongoing pregnancy rate, stillbirth rate, LBR. |
|  |  |  |  |  |  |  |  | DET | 331 | 30.8 ± 3.0 (21.6–35.9) |  |
| Gardner 2004 | Open-label RCT | USA | 24-month | **Included:** D3 FSH ≤ 10 mIU/mL, E2 < 80 pg/mL, hysteroscopically normal endometrial cavity, ≥10 follicles > 12 mm in diameter on day of HCG administration. | Fresh | NA | Blastocyst | SBT | 23 | 33.5 ± 0.9 (26-43) | Implantation rate, ongoing pregnancy rate, MPR. |
|  |  |  |  |  |  |  |  | DBT | 25 | 34.2 ± 0.7 (29-41) |  |
| Davies 2003 (ASSET) | Multi-center RCT | Australian | NA | **Included**: < 35 years if no previous ART pregnancy, < 40 if previous ART pregnancy, ≥4 good-quality embryos or ≥3 if previous ART pregnancy successful. | Fresh | No | Cleavage | SET | 13 | NA | LBR, MBR. |
|  |  |  |  |  |  |  |  | DET | 14 | NA |  |
| Martikainen 2001 | Multi-center RCT | Finland | NA | **Included:** fresh cycle, had/not had ＞1 previous failed treatment, ≥4 good quality embryos, frozen embryo transfers were analysed separately. | Fresh | First/ Second | Cleavage | SET | 74 | 30.8 ± 3.9 (22-38) | CPR, miscarriage rate, ectopic pregnancy rate, LBR, MBR, preterm birth rate, low birthweight rate. |
|  |  |  |  |  |  |  |  | DET | 70 | 30.5 ± 4.1 (21-40) |  |
| Gerris 1999 | Open-label RCT | Belgium | 1997.11-1999.05 | **Included:** < 34 years in their first IVF/ICSI cycle, ≥2 top quality embryos at the time of transfer. | Fresh | Yes | Cleavage | SET | 26 | 31.9 | Positive HCG rate, ongoing pregnancy rate, implantation rate, CPR, MPR, LBR. |
|  |  |  |  |  |  |  |  | DET | 27 |  |  |
| Zhu 2020 | Retro-spective cohort study | China | 2011.01 - 2017.12 | **Included:** women receiving their first FET, used autologous oocytes, and each woman was included only once in this study. **Excluded:** women with previous fresh or FET. | Frozen | Yes | Cleavage/Blastocyst | SET (GQE) | 2991 | NA | LBR, MBR. |
|  |  |  |  |  |  |  |  | SET (PQE) | 510 |  |  |
|  |  |  |  |  |  |  |  | DET (2GQE) | 20980 |  |  |
|  |  |  |  |  |  |  |  | DET (GQE+PQE) | 1807 |  |  |
|  |  |  |  |  |  |  |  | DET (2PQE) | 388 |  |  |
| Su 2020 | Retro-spective cohort study | China | 2017.01 - 2019.04 | **Included:**35-43 years, no contraindications, BMI < 27 kg/m^2^, AFC ≥ 5, b-FSH ≤ 12 IU/L; **Excluded:** husbands had azoospermia, severe oligospermia, asthenozoospermia; endometriosis; adenomyosis; endometrial fibroids; uterine cavity adhesion; endometrial polyps; hydrosalpinx; recurrent miscarriage; chromosomal abnormalities in either of the couples; previous IVF/ICSI failed >2 times. | Fresh | No | Blastocyst | SBT | 84 | 36.8   (35.7, 38.6) | CPR, chemistry pregnancy rate, ectopic pregnancy rate, implantation rate, MPR, miscarriage rate, preterm birth rate, LBR, low-weight infant rate, fetal macrosomia rate, neonatal birth weight. |
|  |  |  |  |  |  |  |  | DBT | 71 | 37.6  (35.9, 40.1) |  |
| Sini 2020 | Retro-spective cohort study | Indonesia | 2015.01 - 2017.12 | **Included:** achieved birth following either a successful eSET or DET;  **Excluded:** with a history of recurrent miscarriages, severe endometriosis, or incomplete infants’ medical records. | Fresh | NA | Blastocyst | eSET | 76 | 31.5 (5.0) | CPR, MPR, LBR, low birth weight rate, preterm birth rate, perinatal mortality rate, Apgar score, NICU admission rate. |
|  |  |  |  |  |  |  |  | DET | 103 | 33.0 (6.0) |  |
| Racca 2020 | Retro-spective, single-center cohort study | Belgium | 2009.01 - 2014.12 | **Included:** only the first frozen embryo transfer of each patient;  **Excluded:** PDG, social freezing, donated cycles. | Frozen | No | Cleavage/Blastocyst | SET | 1936 | 32 (29-36) | CPR, LBR, MBR, positive HCG rate, biochemical pregnancy and early pregnancy loss. |
|  |  |  |  |  |  |  |  | DET | 1665 | 34 (30-38) |  |
| Park 2020 | Retro-spective cohort study | Korea | 2014 - 2015 | **Included:** aged <40 years, with an endometrial thickness ≥7mm; **Excluded:** donor oocytes or blastocysts from other hospitals or solely poor blastocysts, or uterine anoma. | Frozen | No | Blastocyst | DBT (GQE+GQE) | 628 | 34.3 ± 2.7 | Implantation rate, CPR, ectopic pregnancy rate, miscarriage rate, MPR, LBR, MBR, cesarean section rate, gestational ages, birth weight, low birth weight rates, preterm birth rates. |
|  |  |  |  |  |  |  |  | DBT (GQE+PQE) | 401 | 34.6 ± 2.6 |  |
|  |  |  |  |  |  |  |  | SBT (GQE) | 277 | 34.3 ± 2.9 |  |
| Demirel 2020 | Retro-spective cohort study | Turkey | 2010 - 2018 | **Included:**18-42 years, regular menstrual cycles, BMI 19.3–28.9 kg/m^2^, no metabolic or endocrine disorders, normal hormone panel, ICSI cycle with ejaculated sperm, normal uterine cavity, fresh blastocyst transplantation, only one cycle was evaluated; **Excluded:** history of cytotoxic chemo-therapy, radiotherapy, ovarian surgery dehydroepiandrosterone and/or testosterone supplement use. | Fresh | No | Blastocyst | SBT (GQE) | 317 | 31.1 ± 4.8 | Positive HCG rate, biochemical pregnancy rate, miscarriage rate, LBR, MBR, ongoing pregnancy rate. |
|  |  |  |  |  |  |  |  | DBT (GQE+PQE) | 95 | 34.6 ± 5.7 |  |
| Chen 2020 | Retro-spective, single-center study | China | 2016.01 - 2018.10 | **Included:** 20-42 years, basal FSH < 10 mIU/mL, first IVF/ICSI cycle, first FET cycle after whole embryo freezing, endometrium ≥ 7 mm; **Excluded:** donated oocytes or embryos, PGT cycles, known uterine anomalies, untreated hydrosalpinx surgically prior to FET, stage III–IV endometriosis or adenomyosis, uncontrolled endocrine and/ or immune disorders or other systemic diseases. | Frozen | Yes | Blastocyst | SBT (GQE) | 1569 | 29.86 | Implantation rate, CPR, ectopic pregnancy, miscarriage, MPR, LBR, MBR, gestational ages, birth weight, low birth weight rates, preterm birth rate, still birth rate, congenital anomalies rate, gestational days at birth. |
|  |  |  |  |  |  |  |  | DBT (2GQE) | 1113 | 31.22 |  |
|  |  |  |  |  |  |  |  | DBT (GQE+AQE) | 313 | 32.09 |  |
|  |  |  |  |  |  |  |  | DBT (2AQE) | 222 | 30.91 |  |
|  |  |  |  |  |  |  |  | SBT (AQE) | 145 | 30.71 |  |
| Alecsandru 2020 | Pro-spective observational cohort study | Spain | 2014.11 - 2017.11 | **Included:** had either experienced RM (≥2 unexplained miscarriages), or RIF (≥2 failed ART cycles with >4 good-quality embryos transferred); **Excluded:** donated oocytes. | Fresh | No | NA | SET | 38 | 36.9 | Positive pregnancy test rate, biochemical miscarriage rate, miscarriage rate, CPR, LBR. |
|  |  |  |  |  |  |  |  | DET | 27 |  |  |
| Aldemir 2020 | Retro-spective cohort study | Turkey | 2007.01 - 2018.02 | **Included:** ≤ 40 years who had their first, second or third cycles with SET or DET; **Excluded:** with endometrial, uterine pathologies, endometriosis or hydrosalpinx. | Fresh | No | Cleavage/Blastocyst | DET (GQE+GQE) | 498 | 33.5 ± 4.6 | CPR, miscarriage rate, LBR, MPR, preterm delivery rate. |
|  |  |  |  |  |  |  |  | DET (GQE+PQE) | 179 | 34.5 ± 4.3 |  |
|  |  |  |  |  |  |  |  | SET (GQE) | 1621 | 29.2 ± 4.3 |  |
| Zhang 2019 | Pro-spective cohort study | China | 2015.02 - 2017.06 | **Included:** ≤ 42 years, had 3 unsuccessful IVF cycles, had normal ovarian responses to gonadotropic stimulation, exhibited no genetic disease. **Excluded:** PGT, ICSI patients. | Frozen | No | Cleavage/Blastocyst | SBT | 243 | 37.0 ± 5.0 | Implantation rate, CPR, miscarriage rate, ongoing pregnancy rate. |
|  |  |  |  |  |  |  |  | DET | 332 | 36.6 ± 4.9 |  |
| Revelli 2019 | Retro-spective cohort study | Italy | 2016.03 - 2018.07 | **Included:** 28-40 years, BMI 18-25, normal response to gonadotropins, verified normal response to controlled ovarian stimulation, easy transfer of two embryos on D3 or of a single blastocyst on D5. | Fresh | NA | Cleavage/Blastocyst | eSBT | 174 | 34.4 | Implantation rate, CPR, MPR, miscarriage rate, ongoing pregnancy rate. |
|  |  |  |  |  |  |  |  | eDET | 154 | 34.7 |  |
| Park 2019 | Retrospective cohort study | Fertility Center of CHA Gangnam Medical Center (South Korea) | 2014.01-2015.12 | **Included:** aged 35-39 years; **Excluded:** using donor oocytes or solely poor blastocysts or blastocysts from other hospitals, underwent either a natural protocol or an IVM protocol in previous fresh cycles, had endometrial thickness ≤ 7 mm or uterine anomalies. | Frozen | NA | Blastocyst | SBT (GQE) | 102 | 36.6 ± 1.4 | Implantation rate, CPR, MPR, ectopic pregnancy, miscarriage, LBR, birthweight, gestational age. |
|  |  |  |  |  |  |  |  | DBT (GQE+PQE) | 166 | 36.6 ± 1.4 |  |
|  |  |  |  |  |  |  |  | DBT (2GQE) | 166 | 36.6 ± 1.3 |  |
| Jin 2019 | Retro-spective cohort study | China | 2010.01 - 2015.12 | **Included:** ≤ 37 years, with the first assisted reproduction cycle, without a history of hereditary diseases; **Excluded:** PGD/PGS cycles due to chromosomal or other reasons, had no transferrable embryos, received whole-embryo cryopreservation. | Fresh | Yes | Cleavage/Blastocyst | SET | 231 | ≤ 37 | Embryo implantation rate, CPR, abortion rate, MPR, LBR. |
|  |  |  |  |  |  |  |  | DET | 13037 |  |  |
|  |  |  |  |  |  |  |  | SBT | 1623 |  |  |
|  |  |  |  |  |  |  |  | DBT | 116 |  |  |
| Freeman 2019 | Retro-spective cohort study | USA | 2011.01 - 2017.12 | **Included:** < 38 years at the time of oocyte collection, having ≥2 vitrified blastocysts in storage and their first FET during the study time period. | Frozen | No | Blastocyst | eSBT | 387 | 31.8 (23-37) | LBR, MPR, implantation rate. |
|  |  |  |  |  |  |  |  | eDBT | 291 | 31.4 (23-37) |  |
| Tannus 2018 | Retrospective analysis | McGill University Reproductive Centre (Canada); ClinART Fertility Centre, (Turkey) | 2007.09 - 2014.05 | **Included:** PCOS; **Excluded:** couples with severe male factor infertility that needed testicular sperm retrieval or patients with cryptozoospermia, other endocrine disorders. | Fresh | No | Cleavage | SET | 83 | 27.4 ± 3.3 (21-35) | Adjusted LBR. |
|  |  |  |  |  |  |  |  | DET | 76 |  |  |
| Muteshi 2018 | Retro-spective cohort study | UK | 2000.01 - 2014.12 | **Included:** first cycle of ART, confirmed unexplained subfertility or endometriosis, long GnRH agonist; **Excluded:** multiple diagnoses, severe male factor subfertility, donor ART treatment, choosing to freeze embryos or returning for a subsequent cycle. | Fresh | Yes | Cleavage/Blastocyst | SET | 205 | NA | LBR, MBR. |
|  |  |  |  |  |  |  |  | DET | 778 |  |  |
| Mehta 2018 | Retro-spective cohort study | India | 2015.01 - 2015.12 | **Included:** having ≥2 transferable blastocysts; **Excluded:** donor oocytes, had an all freeze cycle due to premature progesterone elevation, the risk of OHSS or unfavorable endometrium. | Fresh | NA | Blastocyst | eSBT | 41 | 34.5 ± 4.3 | Pregnancy rate, implantation rate, CPR, MPR, miscarriage rate, ectopic pregnancy, LBR. |
|  |  |  |  |  |  |  |  | DBT | 123 | 35.0 ± 3.4 |  |
| Dobson 2018 | Pro-spective cohort study | Canada | 2010 - 2016 | **Included:** first cycle of fresh IVF/ICSI or first frozen ET cycle, autologous oocytes, only one cycle per participant; **Excluded:** with gynecological pathologies like fibroids, endometrial polyps, hydrosalpinx, large ovarian cysts, and were not treated before IVF. | Fresh/Frozen | Yes | Blastocyst | SET (GQE) | 613 | 35.3 ± 4.1 | CPR, MPR, LBR, MBR. |
|  |  |  |  |  |  |  |  | SET (PQE) | 131 |  |  |
|  |  |  |  |  |  |  |  | DET (2GQE) | 890 |  |  |
|  |  |  |  |  |  |  |  | DET (GQE+PQE) | 240 |  |  |
|  |  |  |  |  |  |  |  | DET (2PQE) | 71 |  |  |
| Wintner 2017 | Retrospective cohort study | a single university affiliated ART center (Israel) | 2010-2015 | **Included:** IVF and IVF ICSI cycles of fresh non-donor oocytes. | Fresh | NA | Cleavage/Blastocyst | SET (GQE) | 120 | 32.2 ± 5.8 | Implantation rate, CPR, MPR, LBR, miscarriage rate, ectopic pregnancy rate, chemical pregnancy rate. |
|  |  |  |  |  |  |  |  | DET (GQE+PQE) | 180 | 35.1 ± 4.9 |  |
|  |  |  |  |  |  |  |  | DET (2GQE) | 303 | 35.2 ± 4.7 |  |
| Tannus 2017 | Retro-spective cohort study | Turkey | 2012.01 - 2015.06 | **Included:** ≥40 years, have a fresh blastocyst transfer on D5, only one cycle per subject; **Excluded:** with >3 previous IVF cycles, oocyte donation cycles, PGD/PGS cycles, and cycles in which embryos did not reach the blastocyst stage on D5. | Fresh | No | Blastocyst | eSBT | 148 | 40.7 ± 0.8 | Positive pregnancy test rate, CPR, miscarriage rate, LBR, MBR. |
|  |  |  |  |  |  |  |  | DBT | 162 | 41.2 ± 0.9 |  |
| Mersereau 2017 | Retro-spective database analysis | USA | 2004 - 2013 | **Included:** first autologous fresh cycle; **Excluded:** history of prior gonadotropin or IVF treatment, research cycles, embryo banking cycles, or cycles that used a gestational carrier, PGS/PGD cycles. | Fresh | Yes | Cleavage/Blastocyst | SET | 15130 | 33.5 ± 4.2 | LBR, MBR. |
|  |  |  |  |  |  |  |  | SBT | 20256 |  |  |
|  |  |  |  |  |  |  |  | DET | 71179 |  |  |
|  |  |  |  |  |  |  |  | DBT | 74958 |  |  |
| Zhang 2016 | Retro-spective cohort study | China | 2012.01 - 2014.09 | **Included:** with history of cesarean section delivery; **Excluded:** did not receive embryo transfer during the oocyte retrieval cycle. | Fresh | No | Cleavage | eSET | 56 | 34.1 ± 3.9 | Implantation rate, CPR, MPR, miscarriage rate, ectopic pregnancy rate, LBR, MBR, preterm birth rates, birth defect rate, gestational days at birth, birth weight. |
|  |  |  |  |  |  |  |  | DET | 74 | 33.2 ± 4.4 |  |
| Monteleone 2016 | Retro-spective cohort study | Brazil | 2007 - 2015 | **Included:** 18-38 years, who failed to conceive in the fresh eSET, had ≥2 spare good-quality blastocysts that were cryopreserved. | Frozen | No | Blastocyst | eSBT | 40 | 34.5 ± 2.6 | Implantation rate, CPR, MPR. |
|  |  |  |  |  |  |  |  | eDBT | 102 | 34.2 ± 3.4 |  |
| Li 2016 | Retro-spective cohort study | China | 2013.01 - 2014.11 | **Included:** ≤ 40 years, first fresh transfer cycles, ultra-short ovarian stimulation protocols. | Fresh | Yes | Cleavage | SET | 203 | 34.2 ± 5.0 | CPR, miscarriage rate. |
|  |  |  |  |  |  |  |  | DET | 654 | 33.6 ± 5.1 |  |
| Keyhan 2016 | Retro-spective database analysis | USA | 2011 - 2012 | **Included:** ≤ 40 years, first autologous fresh cycle; **Excluded:** GIFT and ZIFT procedures as well as any IVF cycle that was undertaken for the purpose of egg donation, embryo banking, or PGD. | Fresh | Yes | Cleavage/Blastocyst | SET | 2667 | NA | CPR, MPR, LBR, MBR. |
|  |  |  |  |  |  |  |  | DET | 16286 |  |  |
|  |  |  |  |  |  |  |  | SBT | 2461 |  |  |
|  |  |  |  |  |  |  |  | DBT | 7923 |  |  |
| He 2016 | Retro-spective cohort study | China | 2010.01 - 2012.12 | **Included:** ≤ 37 years, ≥ 4 D3 available embryos, did not receive fresh cycle transfer and required whole embryo freezing.  **Exclusion:** not pregnant and still had available embryos before 2014.12.31, requested different numbers of transferred embryos. | Frozen | Yes | Blastocyst | SBT | 383 | 29.1 ± 3.8 | Implantation rate, CPR, MPR, ectopic pregnancy rate, miscarriage rate, early abortion rate. |
|  |  |  |  |  |  |  |  | DBT | 100 | 29.1 ± 3.7 |  |
| Hatırnaz 2016 | Retro-spective cohort study | Turkey | 2007.09 - 2014.05 | **Included:** PCOS patients who underwent an IVM cycle; **Excluded:** Hyperprolactinemia, Cushing syndrome, nonclassic congenital adrenal hyperplasia, adrenal and ovarian androgen secreting tumors. | Fresh | No | Cleavage | SET | 83 | 24.1 ± 4.2 | Implantation rate, CPR, MPR, miscarriage rate, LBR, MBR, perinatal death rate. |
|  |  |  |  |  |  |  |  | BET | 76 | 32.4 ± 3.5 |  |
| Haikin Herzberger 2016 | Retro-spective cohort study | Israel | NA | **Included:** patients in their first, fresh cycle with time lapse microscopy. | Fresh | No | Blastocyst | eSBT | 83 | 29.8 ± 4.9 | CPR, MPR. |
|  |  |  |  |  |  |  |  | DBT | 25 | 34.8 ± 5.0 |  |
| Crawford 2016 | Retro-spective database analysis | USA | 2012 - 2013 | **Included:** <35 years, with no previous ART treatment, fresh, autologous cycles, ≥1 embryo was cryopreserved. | Fresh | Yes | Cleavage/Blastocyst | eSET | 4129 | NA | LBR, MBR. |
|  |  |  |  |  |  |  |  | eDET | 10001 |  |  |
| Mounce 2015 | Secondary analysis of RCT | UK | 2010.03 - 2012.07 | **Included:** < 40 years, had at least one blastocyst or two cleavage-stage embryos in storage; regular ovulatory cycles; and ≤2 previous FER cycles; **Excluded:** participated in the trial more than once. | Frozen | No | Cleavage/Blastocyst | SET | 56 | NA | LBR. |
|  |  |  |  |  |  |  |  | BET | 89 |  |  |
| López Regalado 2014 (b) | Retro-spective cohort study | Spain | 2010.01 - 2013.06 | **Included:** <38 years, BMI 19-29 kg/m^2^, D3 FSH< 15mUI/ml, first cycle IVF/ICSI or second cycle with previous pregnancy not carried to term; **Excluded:** ≥ 5 years of infertility, previous surgery, uterine malformations, repeated miscarriages and previous unsuccessful complete cycles of IVF/ICSI. | Frozen | First/ Second | Cleavage | SET | 101 | 33.5 ± 3.7（21-37） | Implantation rate, miscarriage rate, ongoing pregnancy rate, CPR, MPR, LBR, MBR. |
|  |  |  |  |  |  |  |  | DET | 105 | 34.3 ± 4.1（21-37） |  |
| Ercan 2014 | Retro-spective cohort study | Turkey | 2010.03 - 2011.12 | **Included:** bilateral tubal obstruction, anovulation, pelvic adhesions, endometriosis, unexplained infertility or serious malefactor infertility. | Fresh | No | Cleavage/Blastocyst | SET | 261 | 29.6 ± 4.5 | CPR, MPR, LBR, spontaneus abortion rate. |
|  |  |  |  |  |  |  |  | DET | 92 | 36.5 ± 2.4 |  |
| Chai 2014 | Retro-spective cohort study | China | 2009.10 - 2011.12 | **Included:** ≤ 35 years, undergoing first IVF cycle, endometrial thickness ≥8 mm, had ≥2 good-quality embryos available for transfer or freezing. | Fresh | Yes | Cleavage | eSET | 74 | 31.8 ± 2.0 | Positive pregnancy test rate, CPR, miscarriage rate, ectopic pregnancy rate, LBR, MBR, preterm delivery rate, stillbirth rate, gestation age, birth weight. |
|  |  |  |  |  |  |  |  | DET | 132 | 32.7 ± 2.3 |  |
| Yιlmaz 2013 | Retrospective cohort study | Zekai Tahir Burak Women’s Health Education and Research Hospital (Japan) | 2009.09-2010.09 | **Included:** aged 20-35 years, BMI 18-29 kg/m^2^, regular menstrual cycles, no endometriosis or uterine abnormalities;  **Excluded:** PCOS, history of recurrent pregnancy loss, significant systemic disease or endocrine or metabolic disorder and so on. | Fresh | NA | NA | SET | 281 | 28 (18–34) | CPR, LBR, MPR, miscarriage Rate, cesarean section rate, perinatal fetal morbidity rate, gestational age, birthweight. |
|  |  |  |  |  |  |  |  | DET | 123 | 29 (20–34) |  |
| Wu 2013 | Retro-spective cohort study | China | 2010.04 - 2010.12 | **Included:** primary infertility, ≤ 35 years, FSH level on day 2–3 ≤12 mIU/ml, ≥6 good quality embryos available on day three, cycles simulated by the standard long protocol. | Fresh | NA | Cleavage/Blastocyst | eSBT | 294 | 29.4 ± 2.9 | Implantation rate, CPR, MPR. |
|  |  |  |  |  |  |  |  | DET | 457 | 29.8 ± 2.9 |  |
| Vélez 2013 | Retro-spective cohort study | Canada | 2009, 2010.08 - 2011.08 | **Included:** only the first cycles of stimulated IVF cycle during both periods; **Excluded:** cycles resulting in the cryopreservation of all embryos. | Fresh | No | Cleavage/Blastocyst | SET | 639 | NA | MPR. |
|  |  |  |  |  |  |  |  | DET | 343 |  |  |
| Niinimäki 2013 | Retro-spective cohort study | Finland | 2000 - 2009 | **Included:** 40-44 years, data on the fresh and frozen embryo transfers following one oocyte retrieval; **Excluded:** donor oocytes. | Fresh | NA | Cleavage | eSET | 264 | 40.8 (41.0) | CPR, LBR, MPR, ectopic pregnancy rate, miscarriage rate. |
|  |  |  |  |  |  |  |  | DET | 364 | 41.1 (41.0) |  |
| Bastu 2013 | Retro-spective cohort study | Turkey | 2010.03 - 2012.01 | **Included:** ≤ 41 years, non-smoking, had normal baseline ovarian reserve testing, a normal uterine cavity based on hysterosalpingogram and/or sonohysterogram, no evidence of hydrosalpinx on pelvic sonogram, underwent GnRH antagonist protocol and used freshly ejaculated sperm; **Excluded:** had incomplete data. | Fresh | No | Cleavage | SET | 263 | NA | Positive HCG rate, CPR. |
|  |  |  |  |  |  |  |  | DET | 117 |  |  |
| Virro 2012 | Retro-spective cohort study | Canada | 1999.01 - 2011.12 | **Included:** with multiple failures and/or unexplained infertility; **Excluded:** donor egg and frozen egg cycles. | Fresh | No | Cleavage/Blastocyst | SET | 33 | 34.9 ± 3.8 | Positive HCG rate, LBR. |
|  |  |  |  |  |  |  |  | DET | 160 | 34.7 ± 3.7 |  |
| Rodríguez Barredo 2012 | Pro-spective cohort study | Spain | 2002.10 - 2006.12 | **Included:** < 30 years with ≥1 GQE; between 30–32 years old with ≥2 GQE and between 33–34 years with ≥3 GQE. | Fresh | NA | Cleavage | SET | 328 | 30.8 ± 0.5 | Implantation rate, CPR, MPR, miscarriage rate. |
|  |  |  |  |  |  |  |  | DET | 300 | 30.9 ± 0.9 |  |
| Gremeau 2012 | Retro-spective cohort study | France | 2005.01 - 2008.12 | **Included:** ≤ 36 years, at their first and second IVF/ICSI cycles and had two embryos with ≥1 good quality observed at D2; **Excluded:** only one embryo or having no transfer, having received a transfer of more than 2 embryos. | Fresh | Yes (extract first cycle data) | Cleavage | eSET | 413 | NA | Ongoing pregnancy rate, LBR, MBR. |
|  |  |  |  |  |  |  |  | DET | 198 |  |  |
| Guerif 2011 | Pro-spective cohort study | France | 2007.01-2009.07 | **Included:** ≤ 36 years, couples attempting ﬁrst or second IVF, without TQE on D2, couples were included only once. | Fresh | First/ Second | Cleavage/Blastocyst | eSBT | 198 | 32.4 ± 4.2 (23-35) | CPR, LBR, MBR, clinical implantation rate, miscarriage rate, ectopic pregnancy rate. |
|  |  |  |  |  |  |  |  | DET | 225 | 32.1 ± 4.6 (21-35) |  |
| Friedman 2011 | Retro-spective cohort study | USA | A ﬁve-year period | **Included:** fresh nondonor cycles in which patients electively chose to transfer one or two blastocysts. | Fresh | NA | Blastocyst | eSBT | 108 | 33.9 ± 3.2 | CPR, MPR, LBR, cesarean rate, birth weight. |
|  |  |  |  |  |  |  |  | DBT | 415 | 35 ± 3.6 |  |
| Bissonnette 2011 | Retro-spective cohort study | Canada | 2010.08-2010.10 | **Included:** all IVF cycles started in Quebec assisted reproduction centers from 2010.08.05-2010.11.05 were recorded. | Fresh | NA | Cleavage/Blastocyst | SET | 707 | 37 (22-46) | CPR, MPR. |
|  |  |  |  |  |  |  |  | DET | 344 |  |  |
| Barri 2011 | Pro-spective cohort study | Spain | NA | **Included:** < 38 years, ≥2 good quality embryos. | Fresh | No | NA | eSET | 355 | NA | CPR, implantation rate, MPR, LBR, miscarriage rate, preterm birth rates, low birth weight rates. |
|  |  |  |  |  |  |  |  | DET | 325 | NA |  |
| Wang 2010 | Retro-spective population-based study | Australia | 2004.01-2007.12 | **Included:** ≥ 18 years, autologous cycles, ﬁrst stimulated fresh cycle using follicle stimulating hormone; **Excluded:** mixed cleavage-blastocyte stage embryo transfer cycles. | Fresh | Yes | Cleavage/Blastocyst | SET | 21341 | NA | CPR, LBR, healthy baby rate. |
|  |  |  |  |  |  |  |  | DET | 12694 | NA |  |
| Sato 2010 | Retro-spective cohort study | Japan | 2005.01-2008.12 | **Included:** ≤ 37 years, previous IVF/ICSI trials ≤ 5, acquisition of ≥2 good-quality embryos. | Fresh/Frozen | No | Cleavage/Blastocyst | eSET | 159 | 33.6 ± 0.4 | CPR, MPR, miscarriage rate, LBR. |
|  |  |  |  |  |  |  |  | DET | 97 | 34.8 ± 0.4 |  |
|  |  |  |  |  |  |  |  | eDET | 112 | 33.8 ± 0.4 |  |
| Nakagawa 2010 | Retro-spective cohort study | Japan | 2005.01-2008.12 | **Included:** first ART treatment, DET was mainly carried out between 2005.01-2008.03, and SET was carried out between 2008.04 -2008.12 | Fresh | Yes | Cleavage | SET | 102 | 34.3 ± 0.4 | CPR, miscarriage rate, ongoing pregnancy rate, LBR, MPR. |
|  |  |  |  |  |  |  |  | DET | 583 | 35.7 ± 0.2 |  |
| Fauque 2010 | Pro-spective nonrandomized study | France | 2005 - 2007 | **Included:** < 36 years, with adequate ovarian function, in their ﬁrst or second IVF or ICSI attempt, with at least 4 mature oocytes and 2 fertilized top quality embryos, patients who refused e-SET had two top embryos. | Fresh | First/ Second | Cleavage | eSET | 53 | 31.2 ± 0.4 (22-36) | CPR, implantation rate, ectopic pregnancy rate, MPR, miscarriage rate, LBR, MBR, gestational weeks, preterm birth rates, birthweight, low birth weight rates, NICU admission rate, perinatal mortality rate. |
|  |  |  |  |  |  |  |  | DET | 98 | 31.6 ± 0.3 (23-36) |  |
| Sundström 2009 | Retro-spective cohort study | Sweden | 2002.07-2004.06 | **Included:** completion of three ET cycles offered to the patients or delivery after the first or second ET cycle. DET was performed in cases in which the embryos were of suboptimal quality for transfer. | Fresh | Yes (extract first cycle data) | Cleavage | SET | 352 | NA | LBR. |
|  |  |  |  |  |  |  |  | eSET | 263 | NA |  |
|  |  |  |  |  |  |  |  | Non-eSET | 89 | NA |  |
|  |  |  |  |  |  |  |  | DET | 18 | NA |  |
| Roberts 2009 | Retro-spective cohort study | UK | 1998-2003 | **Included:** < 40 years, no children aged ≤ 16 years living together, stable co-habitation, the earliest available treatment cycle in the period from each patient; **Excluded**: abandoned cycles, patients with incomplete information. | Fresh | No | Cleavage | SET | 182 | NA | LBR, MBR. |
|  |  |  |  |  |  |  |  | DET | 872 | NA |  |
| Bechoua 2009 | Retro-spective + Pro-spective cohort study | France | 2001.01-2007.12 | **Included:** < 35 years at the time of the transfer of fresh embryos, ﬁrst IVF cycle, had ≥3 embryos of good quality available for transfer or freezing; **Excluded:** patients for whom eSET was requested for medical reasons. | Fresh | Yes | Cleavage | SET | 243 | 29.7 ± 3.2 | CPR, implantation rate, LBR, MBR. |
|  |  |  |  |  |  |  |  | DET | 483 | 29.6 ± 3.1 |  |
| Van Peperstraten 2008 | Retro-spective cohort study | The Netherlands | 2005 | **Included:** first IVF/ICSI cycle ever or a first cycle after a successful IVF/ICSI treatment, aged < 40 years at ovum pick-up, ≥ 2 embryos available, with ≥1 embryo of excellent or good quality (no or <10% fragmentation). | Fresh | No | Cleavage | eSET | 187 | 32.4 (26-38) | Ongoing pregnancies, MPR. |
|  |  |  |  |  |  |  |  | DET | 290 | 33 (24-40) |  |
| Styer 2008 | Retro-spective cohort study | USA | 2002.01-2006.03 | **Included:** fresh blastocyst transfer, ≤37 years, ≥5 embryos at the six- to eight-cell stage on day 3. | Fresh | No | Blastocyst | eSBT | 52 | 31.4 ± 3.6 | Positive serum HCG rate, CPR, LBR, pregnancy loss rate, implantation rate, ectopic pregnancy rate, MPR. |
|  |  |  |  |  |  |  |  | DBT | 187 | 32.0 ± 3.5 |  |
| Kalu 2008 | Pro-spective cohort study | UK | 2005.01-2006.12 | **Included:** 700 cycles of IVF/ICSI involving fresh blastocyst transfer and a total of 102 cycles of FBT performed in women aged 25–43 years. | Fresh | Yes | Blastocyst | SBT | 280 | 25-43 | CPR, MPR, LBR, MBR. |
|  |  |  |  |  |  |  |  | DBT | 420 |  |  |
| Lundin 2007 | Retro-spective cohort study | Sweden | 2003-2004 | **Included:** all patients receiving their ﬁrst fresh transfer in 2003 or 2004 were included in this study | Fresh | Yes | Cleavage | SET | 531 | 32.2 ± 3.8 (21.9–40.2) | LBR. |
|  |  |  |  |  |  |  |  | DET | 158 | 34.0 ± 3.5 (24.4–42.1) |  |
| Donoso 2007 | Retro-spective cohort study | Belgium | 2001.01-2005.06 | **Included:** < 36 years, ﬁrst treatment cycle, patients had undergone embryo transfer. | Fresh | Yes | Blastocyst | SBT | 62 | 29.8 ± 3.2 | MBR, LBR. |
|  |  |  |  |  |  |  |  | DBT | 73 | 29.8 ± 2.9 |  |
| Veleva 2006 | Retro-spective cohort study | Finland | 2000-2003 | **Included:** 1224 fresh embryo transfers were carried out in women aged 36–39 years. | Fresh | No | Cleavage | eSET | 335 | 37.5 ± 1.1 | CPR, LBR, MPR, miscarriage rate. |
|  |  |  |  |  |  |  |  | nt-eSET | 110 | 37.3 ± 1.0 |  |
|  |  |  |  |  |  |  |  | cSET | 194 | 37.6 ± 1.1 |  |
|  |  |  |  |  |  |  |  | DET | 585 | 37.6 ± 1.1 |  |
| van Montfoort 2006 | Cohort of a randomized patient-blind trial | The Netherlands | 2002.01-2004.12 | **Included:** first treatment cycle, ≥2 normally fertilized oocytes had to be present; **Excluded:** PGD, eSET for medical reasons. | Fresh | Yes | Cleavage | eSET | 100 | NA | Positive HCG rate, abortion rate, ongoing pregnancy rate, MPR, miscarriage rate. |
|  |  |  |  |  |  |  |  | DET | 122 | NA |  |
| Le Lannou 2006 | Pro-spective cohort study | France | 2002.06-2004.12 | **Included:** ﬁrst cycle of treatment, < 38 years, had ≥2 good quality embryos. | Fresh | Yes | Cleavage | SET | 130 | 30.1  (21–37) | LBR, CPR, implantation rate, LBR, MBR, ectopic pregnancy rate, miscarriage rate. |
|  |  |  |  |  |  |  |  | DET | 130 | 31.8  (24–37) |  |
| Saldeen 2005 | Retro-spective cohort study | Sweden | 2003.01-2003.09 | **Included:** ET was in force, and DET was allowed under certain circumstances (poor embryo quality, > 39 years, ≥ 3 previously failed ET cycles). | Fresh | No | Cleavage | SET | 315 | NA | CPR, ongoing pregnancy rate, MPR. |
|  |  |  |  |  |  |  |  | DET | 118 | NA |  |
| Henman 2005 | Pro-spective cohort study | Australia | 2000.04-2004.04 | **Included:** ＜38 years, ≥3 usable blastocyst, recruited from 2000.04 -2001.12; **Excluded:**  IVF and PGD. | Fresh | NA | Blastocyst | SBT | 121 | NA | CPR, implantation rate, MPR, miscarriage rate, LBR. |
|  |  |  |  |  |  |  |  | DBT | 285 | NA |  |
| Hara 2005 | Retro-spective cohort study | Japan | 2001.12-2003.12 | **Included:** undergoing conventional IVF in the department during this period; **Excluded:** cycles with ICSI or frozen and thawed embryos. | Fresh | NA | Blastocyst | SBT | 16 | 34.3 ± 0.5 (26-42) | CPR, implantation rate, MPR, miscarriage rate. |
|  |  |  |  |  |  |  |  | DBT | 48 |  |  |
| Criniti 2005 | Retro-spective cohort study | USA | 2003.01-2004.08 | **Included:** < 38 years or, no history of failed IVF cycles, no moderate or severe endometriosis, trilaminar endometrium > 7 mm on day of HCG administration, normal uterine cavity and so on. | Fresh | NA | Blastocyst | SBT | 25 | 23-37 | CPR, implantation rate, MPR. |
|  |  |  |  |  |  |  |  | DBT | 46 |  |  |
| Martikainen 2004 | Retro-spective cohort study | Finland | 2000-2002 | **Included:** SET is routinely carried out among women <36 years in the first or second treatment cycle when a top-quality embryo is available. | Fresh | First/ Second | Cleavage | eSET | 468 | 21-43 | CPR. |
|  |  |  |  |  |  |  |  | DET | 803 |  |  |
| Kuramoto 2004 | Retro-spective cohort study | Japan | 1998.08-2002.07 | **Included:** < 40 years using the conventional IVF and ICSI procedures. | Fresh | NA | Blastocyst | SBT | 60 | NA | CPR, implantation rate, MPR, miscarriage rate. |
|  |  |  |  |  |  |  |  | DBT | 200 | NA |  |
| Gerris 2004 | Pro-spective cohort study | Belgium | 2000.01-2001.12 | **Included:** <38 years at the time of embryo transfer, first IVF/ICSI treatment ever or after a previous delivery; **Excluded:** no embryos available or no embryos of sufficient quality to transfer. | Fresh | No | Cleavage | SET | 206 | 30.9 ± 3.6 | Ongoing pregnancy rate, LBR, MBR. |
|  |  |  |  |  |  |  |  | DET | 161 |  |  |
| De Neubourg 2002 | Retro-spective cohort study | Belgium | 2000.01-2001.12 | **Included:** < 38 years old in the ﬁrst IVF/ICSI cycle or after a previous delivery. | Fresh | No | Cleavage | SET | 127 | 31.3 ± 3.3 | Singleton pregnancy rate, MPR, ongoing pregnancy rate, implantation rate. |
|  |  |  |  |  |  |  |  | DET | 116 | 30.4 ± 3.9 |  |
| Martikainen 2001 | Pro-spective cohort study | Finland | NA | **Included:** had ≥4 good quality embryos who were undergoing their first or second cycle. | Fresh | First/ Second | Cleavage | SET | 187 | NA | CPR, LBR, MBR. |
|  |  |  |  |  |  |  |  | DET | 970 | NA |  |
| Rutherford 1988 | Pro-spective cohort study | UK | NA | **Included:** infertile women were given buserelin or clomiphene citrate followed by induction of ovulation by human menopausal gonadotrophin. | Fresh | NA | Cleavage | SET | 45 | NA | CPR. |
|  |  |  |  |  |  |  |  | DET | 54 | NA |  |
| Speirs 1983 | Retro-spective cohort study | Australia | 1982 | **Included:** the first 170 patients having embryo transfer in 1982. | Fresh | NA | NA | SET | 60 | NA | CPR. |
|  |  |  |  |  |  |  |  | DET | 49 | NA |  |
|  |  |  |  |  |  |  |  |  |  |  |  |
| Note: Abbreviation: AFC, antral follicle count; ART, assisted reproductive technology; b-FSH, basal follicle-stimulating hormone; BMI, body Mass Index; CPR, clinical pregnancy rate; DBT, double blastocyst transfer; DET, double embryo transfer; E2, estradiol; ET, embryo transfer; FBT, frozen blastocyst transfer; FER, frozen embryo replacement; FET, frozen embryo transfer; FSH, follicle-stimulating hormone; GIFT, gamete Intra Fallopian Transfer; GQE, good quality embryo; HCG, human Chorionic Gonadotropin; ICSI, intracytoplasmic sperm injection; IR, implantation rate; IVF, in-vitro fertilization; IVM, in vitro maturation; LBR, live birth rate; MBR, multiple live birth rate; MPR, multiple pregnancy rate; NA, not applicable or not reported; OHSS, ovarian hyperstimulation syndrome; PCOS, polycystic ovary syndrome; PGD, preimplantation genetic diagnosis; PGS, preimplantation genetic screening; PGT, preimplantation genetic testing; POR, poor ovarian response; PQE, poor quality embryo; RIF, recurrent implantation failure; RM, recurrent miscarriage; SBT, single blastocyst transfer; SET, single embryo transfer; TQE, top quality embryo; ZIFT, zygote intrafallopian transfer. | | | | | | | | | | | |
|  |  |  |  |  |  |  |  |  |  |  |  |
|  |  |  |  |  |  |  |  |  |  |  |  |
|  |  |  |  |  |  |  |  |  |  |  |  |

| **Supplementary Table S2. Methodological quality of included studies.** | | | | | | | |
| --- | --- | --- | --- | --- | --- | --- | --- |
| **RCTs** | **Cochrane risk of bias tool** | | | | | | |
|  | Random sequence generation (selection bias) | Allocation concealment (selection bias) | Blinding for participants and personnel (performance bias) | Blinding of outcome assessment (detection bias) | Incomplete outcome data (attrition bias) | Selective reporting (reporting bias) | Other bias |
| Abuzeid, O. M. 2017 | low | low | high | low | low | low | high |
| Prados, N. 2015 | low | low | high | low | low | low | high |
| López-Regalado, M. L. 2014 | low | high | high | low | low | low | low |
| Forman, E. J. 2013 | low | low | high | low | low | low | high |
| Moustafa, M. K. 2008 | unclear | unclear | low | low | low | low | high |
| van Montfoort, A. P. 2006 | unclear | unclear | low | low | low | low | unclear |
| Bhattacharya 2006 | low | low | low | low | low | low | unclear |
| Thurin 2005 | low | unclear | low | low | low | low | unclear |
| Lukassen, H. G. M. 2005 | low | unclear | high | low | low | low | low |
| Thurin, A. 2004 / Kjellberg, A. T. 2006 | low | unclear | low | low | low | low | unclear |
| Gardner, D. K. 2004 | low | unclear | high | low | unclear | unclear | unclear |
| ASSET2003 | low | low | low | low | low | low | unclear |
| Martikainen, H. 2001 | low | unclear | high | low | low | low | unclear |
| Gerris, J. 1999 | low | unclear | high | low | low | low | unclear |
|  |  |  |  |  |  |  |  |
| **Observational studies** | **Newcastle-Ottawa Scale (NOS)** | | | | | | |
|  | Selection | | Comparability | | Outcome | | Overall quality |
| Zhu, Q. 2020 | 4 | | 1 | | 3 | | 8 |
| Su, W. 2020 | 3 | | 0 | | 3 | | 6 |
| Sini, I. 2020 | 3 | | 1 | | 2 | | 6 |
| Racca, A. 2020 | 4 | | 1 | | 3 | | 8 |
| Park, D. S. 2020 | 4 | | 1 | | 3 | | 8 |
| Demirel, C. 2020 | 4 | | 0 | | 3 | | 7 |
| Chen, S. 2020 | 4 | | 0 | | 2 | | 6 |
| Alecsandru, D. 2020 | 3 | | 0 | | 3 | | 6 |
| Aldemir, O. 2020 | 4 | | 0 | | 3 | | 7 |
| Zhang, X. 2019 | 3 | | 1 | | 3 | | 7 |
| Revelli, A. 2019 | 4 | | 0 | | 3 | | 7 |
| Park 2019 | 3 | | 1 | | 3 | | 7 |
| Jin, H. X. 2019 | 4 | | 0 | | 3 | | 7 |
| Freeman, M. R. 2019 | 4 | | 0 | | 3 | | 7 |
| Tannus, S. 2018 | 4 | | 1 | | 3 | | 8 |
| Muteshi, C. M. 2018 | 4 | | 0 | | 3 | | 7 |
| Mehta, V. 2018 | 4 | | 0 | | 3 | | 7 |
| Dobson, S. J. A. 2018 | 4 | | 0 | | 3 | | 7 |
| Wintner 2017 | 4 | | 0 | | 3 | | 7 |
| Tannus, S. 2017 | 4 | | 1 | | 3 | | 8 |
| Mersereau, J. 2017 | 4 | | 0 | | 3 | | 7 |
| Zhang, N. 2016 | 3 | | 0 | | 3 | | 6 |
| Monteleone, P. A. 2016 | 4 | | 1 | | 3 | | 8 |
| Li, M. 2016 | 4 | | 0 | | 3 | | 7 |
| Keyhan, S. 2016 | 4 | | 0 | | 3 | | 7 |
| He, Q. H. 2016 | 4 | | 0 | | 3 | | 7 |
| Hatırnaz, S. 2016 | 4 | | 1 | | 3 | | 8 |
| Haikin Herzberger, E. 2016 | 4 | | 0 | | 3 | | 7 |
| Crawford, S. 2016 | 4 | | 0 | | 3 | | 7 |
| Mounce, G. 2015 | 4 | | 0 | | 3 | | 7 |
| López Regalado, M. L. 2014 | 4 | | 0 | | 3 | | 7 |
| Ercan, C. M. 2014 | 4 | | 0 | | 3 | | 7 |
| Chai, J. 2014 | 4 | | 0 | | 2 | | 6 |
| Wu, K. L. 2013 | 4 | | 0 | | 3 | | 7 |
| Yιlmaz 2013 | 4 | | 0 | | 3 | | 7 |
| Vélez, M. P. 2013 | 4 | | 0 | | 3 | | 7 |
| Niinimäki, M. 2013 | 4 | | 0 | | 2 | | 6 |
| Bastu, E. 2013 | 4 | | 0 | | 3 | | 7 |
| Virro, M. R. 2012 | 4 | | 0 | | 3 | | 7 |
| Rodríguez Barredo, D. B. 2012 | 4 | | 0 | | 3 | | 7 |
| Gremeau, A. S. 2012 | 4 | | 0 | | 3 | | 7 |
| Guerif, F. 2011 | 4 | | 0 | | 3 | | 7 |
| Friedman, B. E. 2011 | 4 | | 1 | | 3 | | 8 |
| Bissonnette, F. 2011 | 4 | | 1 | | 3 | | 8 |
| Barri, P. N. 2011 | 4 | | 0 | | 3 | | 7 |
| Wang, Y. A. 2010 | 4 | | 1 | | 3 | | 8 |
| Sato, W. 2010 | 4 | | 0 | | 3 | | 7 |
| Nakagawa, K. 2010 | 4 | | 0 | | 3 | | 7 |
| Fauque, P. 2010 | 4 | | 0 | | 3 | | 7 |
| Sundström, P. 2009 | 4 | | 0 | | 3 | | 7 |
| Roberts, S. A. 2009 | 4 | | 1 | | 3 | | 8 |
| Bechoua, S. 2009 | 4 | | 1 | | 3 | | 8 |
| Van Peperstraten, A. M. 2008 | 4 | | 0 | | 3 | | 7 |
| Styer, A. K. 2008 | 4 | | 0 | | 3 | | 7 |
| Kalu, E. 2008 | 4 | | 0 | | 3 | | 7 |
| Lundin, K. 2007 | 4 | | 0 | | 3 | | 7 |
| Donoso, P. 2007 | 4 | | 0 | | 3 | | 7 |
| Veleva, Z. 2006 | 4 | | 0 | | 3 | | 7 |
| van Montfoort, A. P. 2006 | 4 | | 0 | | 3 | | 7 |
| Le Lannou, D. 2006 | 4 | | 1 | | 3 | | 8 |
| Saldeen, P. 2005 | 4 | | 0 | | 3 | | 7 |
| Henman, M. 2005 | 4 | | 0 | | 3 | | 7 |
| Hara, T. 2005 | 3 | | 0 | | 3 | | 6 |
| Criniti, A. 2005 | 3 | | 0 | | 3 | | 6 |
| Martikainen, H. 2004 | 4 | | 0 | | 3 | | 7 |
| Kuramoto, T. 2004 | 4 | | 1 | | 3 | | 8 |
| Gerris, J. 2004 | 4 | | 0 | | 3 | | 7 |
| De Neubourg, D. 2002 | 4 | | 1 | | 3 | | 8 |
| Martikainen, H.2001 | 4 | | 0 | | 3 | | 7 |
| Rutherford, A. J. 1988 | 4 | | 0 | | 3 | | 7 |
| Speirs, A. L. 1983 | 4 | | 0 | | 3 | | 7 |

| **Supplementary Table S3. Sensitivity and subgroup analyses comparing clinical pregnancy rate after SET and DET in a single cycle.** | | | | | | |
| --- | --- | --- | --- | --- | --- | --- |
|  | **Studies no.** | **SET total** | **DET total** | ***I^2^*** | **OR (95%CI)** | ***P* value** |
| **Overall** | 60 | 43414 | 68289 | 82% | 0.78 (0.71-0.85) | <0.001 |
| **Subgroup analyses** | | | | | |  |
| Maternal age (years) |  |  |  |  |  | 0.483^*^ |
| <35^#^ | 16 | 17823 | 18180 | 87% | 0.72 (0.59-0.87) | 0.001 |
| 35-40 | 7 | 11238 | 20431 | 65% | 0.82 (0.71-0.94) | 0.004 |
| ≥40 | 5 | 2666 | 3546 | 70% | 0.88 (0.59-1.30) | 0.508 |
| Quality |  |  |  |  |  | - |
| G/GG | 10 | 4628 | 3921 | 14% | 0.61 (0.55-0.68) | <0.001 |
| G/GP | 8 | 4541 | 1447 | 0% | 1.01 (0.89-1.16) | 0.833 |
| P/PP | 6 | 464 | 529 | 0% | 0.74 (0.55-0.99) | 0.044 |
| Embryo stage |  |  |  |  |  | 0.548^*^ |
| Cleavage | 28 | 22613 | 47875 | 84% | 0.70 (0.61-0.81) | <0.001 |
| Blastocyst | 26 | 17911 | 17594 | 86% | 0.75 (0.64-0.88) | 0.001 |
| Cycle |  |  |  |  |  | 0.975^*^ |
| Fresh | 48 | 37558 | 61665 | 82% | 0.78 (0.71-0.87) | <0.001 |
| Frozen | 9 | 5236 | 5882 | 83% | 0.79 (0.62-1.00) | 0.052 |
| Design |  |  |  |  |  | 0.010^*^ |
| RCT | 8 | 516 | 517 | 0% | 0.55 (0.43-0.72) | <0.001 |
| Observational study | 52 | 42898 | 67772 | 83% | 0.80 (0.73-0.88) | <0.001 |
| **Sensitivity analyses** | | | | | |  |
| Adjusted | 6 | 948 | 2247 | 80% | 0.85 (0.61-1.18) | 0.326 |
| First cycle | 14 | 32330 | 55693 | 94% | 0.68 (0.57-0.80) | <0.001 |
| eSET | 35 | 19435 | 14736 | 65% | 0.82 (0.73-0.92) | 0.001 |
| Note:  #, included two studies (Chai 2014 and Wu 2013) whose participants aged less than or equal to 35 years. Abbreviation: CI, confidence interval; DET, double embryo transfer; eSET, elective single embryo transfer; G/GG, a single good quality embryo (GQE) compared with two GQEs; G/GP, a single GQE compared with two embryos of mixed quality (GQE+PQE); OR, odds ratio; P/PP, a single non-top quality embryo (PQE) compared with two PQEs; RCT, randomized controlled trial; SET, single embryo transfer.  ^*^, P for interaction. | | | | | | |

| **Supplementary Table S4. Sensitivity and subgroup analyses comparing miscarriage rate after SET and DET in a single cycle.** | | | | | | |
| --- | --- | --- | --- | --- | --- | --- |
|  | **Studies no.** | **SET total** | **DET total** | ***I^2^*** | **OR (95%CI)** | ***P* value** |
| **Overall** | 43 | 5230 | 13249 | 30% | 1.10 (0.95-1.27) | 0.211 |
| **Subgroup analyses** | | | | | |  |
| Maternal age (years) |  |  |  |  |  | 0.912^*^ |
| <35^#^ | 6 | 1325 | 1254 | 0% | 1.13 (0.90-1.42) | 0.290 |
| 35-40 | 3 | 238 | 382 | 0% | 1.06 (0.69-1.63) | 0.775 |
| ≥40 | 2 | 111 | 124 | 0% | 1.24 (0.73-2.11) | 0.435 |
| Quality |  |  |  |  |  | - |
| G/GG | 7 | 1813 | 1672 | 47% | 1.11 (0.79-1.57) | 0.546 |
| G/GP | 7 | 1955 | 650 | 3% | 1.06 (0.80-1.41) | 0.673 |
| P/PP | 3 | 69 | 139 | 12% | 1.68 (0.48-5.83) | 0.417 |
| Embryo stage |  |  |  |  |  | 0.408^*^ |
| Cleavage | 19 | 1189 | 9222 | 49% | 1.23 (0.90-1.68) | 0.187 |
| Blastocyst | 17 | 3166 | 3024 | 18% | 1.05 (0.86-1.29) | 0.628 |
| Cycle |  |  |  |  |  | 0.969^*^ |
| Fresh | 35 | 3497 | 10884 | 39% | 1.13 (0.94-1.36) | 0.206 |
| Frozen | 6 | 1607 | 2177 | 0% | 1.12 (0.92-1.37) | 0.255 |
| Design |  |  |  |  |  | 0.229^*^ |
| RCT | 8 | 381 | 522 | 42% | 1.46 (0.88-2.44) | 0.146 |
| Observational study | 35 | 4849 | 12727 | 26% | 1.05 (0.91-1.22) | 0.491 |
| **Sensitivity analyses** | | | | | |  |
| Adjusted | 3 | 221 | 763 | 32% | 1.28 (0.91-1.78) | 0.540 |
| First cycle | 10 | 2479 | 9712 | 40% | 1.43 (1.10-1.86) | 0.008 |
| eSET | 27 | 1711 | 2450 | 30% | 1.14 (0.92-1.41) | 0.221 |
| Note:  ^#^, included one study (Chai 2014) whose participants aged less than or equal to 35 years. Abbreviation: CI, confidence interval; DET, double embryo transfer; eSET, elective single embryo transfer; G/GG, a single good quality embryo (GQE) compared with two GQEs; G/GP, a single GQE compared with two embryos of mixed quality (GQE+PQE); OR, odds ratio; P/PP, a single non-top quality embryo (PQE) compared with two PQEs; RCT, randomized controlled trial; SET, single embryo transfer. ^*^, P for interaction. | | | | | | |
|  |  |  |  |  |  |  |
|  |  |  |  |  |  |  |
|  |  |  |  |  |  |  |
|  |  |  |  |  |  |  |
|  |  |  |  |  |  |  |
|  |  |  |  |  |  |  |
|  |  |  |  |  |  |  |

| **Supplementary Table S5. Sensitivity and subgroup analyses comparing preterm birth rate after SET and DET in a single cycle.** | | | | | | |
| --- | --- | --- | --- | --- | --- | --- |
|  | **Studies no.** | **SET total** | **DET total** | ***I^2^*** | **OR (95%CI)** | ***P* value** |
| **Overall^§^** | 13 | 1852 | 2380 | 0% | 0.25 (0.21-0.30) | <0.001 |
| **Subgroup analyses** | | | | | |  |
| Maternal age (years) |  |  |  |  |  | 0.832^*^ |
| <35^#^ | 4 | 884 | 877 | 27% | 0.28 (0.16-0.50) | <0.001 |
| ≥35 | 3 | 131 | 404 | 0% | 0.25 (0.13-0.48) | <0.001 |
| Quality |  |  |  |  |  | - |
| G/GG | 4 | 1429 | 1267 | 0% | 0.22 (0.18-0.28) | <0.001 |
| G/GP | 4 | 1427 | 422 | 0% | 0.32 (0.23-0.44) | <0.001 |
| P/PP | 1 | 50 | 101 | - | 0.10 (0.03-0.33) | <0.001 |
| Embryo stage |  |  |  |  |  | 0.231^*^ |
| Cleavage | 6 | 325 | 306 | 0% | 0.34 (0.20-0.57) | <0.001 |
| Blastocyst | 7 | 1394 | 1925 | 0% | 0.24 (0.19-0.29) | <0.001 |
| Cycle |  |  |  |  |  | 0.072^*^ |
| Fresh | 10 | 824 | 738 | 0% | 0.32 (0.23-0.43) | <0.001 |
| Frozen | 3 | 1028 | 1642 | 0% | 0.22 (0.18-0.28) | <0.001 |
| Design |  |  |  |  |  | 0.576^*^ |
| RCT | 3 | 60 | 82 | 0% | 0.33 (0.12-0.90) | 0.031 |
| Observational study | 10 | 1792 | 2298 | 0% | 0.25 (0.21-0.30) | <0.001 |
| **Sensitivity analyses** | | | | | |  |
| Adjusted | 3 | 221 | 696 | 0% | 0.29 (0.20-0.43) | <0.001 |
| First cycle | 2 | 913 | 1047 | 63% | 0.31 (0.10-0.95) | 0.040 |
| eSET | 6 | 306 | 455 | 0% | 0.33 (0.23-0.49) | <0.001 |
| Note:  ^§^, preterm birth rate was calculated as the number of preterm births divided by the total number of live births (multiple gestations included) in one of included studies (Sini 2020). ^#^, included one study (Chai 2014) whose participants aged less than or equal to 35 years. Abbreviation: CI, confidence interval; DET, double embryo transfer; eSET, elective single embryo transfer; G/GG, a single good quality embryo (GQE) compared with two GQEs; G/GP, a single GQE compared with two embryos of mixed quality (GQE+PQE); OR, odds ratio; P/PP, a single non-top quality embryo (PQE) compared with two PQEs; RCT, randomized controlled trial; SET, single embryo transfer.  ^*^, P for interaction. | | | | | | |
|  |  |  |  |  |  |  |
|  |  |  |  |  |  |  |
|  |  |  |  |  |  |  |
|  |  |  |  |  |  |  |
|  |  |  |  |  |  |  |
|  |  |  |  |  |  |  |
|  |  |  |  |  |  |  |

| **Supplementary Table S6. Sensitivity and subgroup analyses comparing low birth weight rate after SET and DET in a single cycle.** | | | | | | |
| --- | --- | --- | --- | --- | --- | --- |
|  | **Studies no.** | **SET total** | **DET total** | ***I^2^*** | **OR (95%CI)** | ***P* value** |
| **Overall** | 9 | 1360 | 2826 | 0% | 0.20 (0.16-0.25) | <0.001 |
| **Subgroup analyses** | | | | | |  |
| Maternal age (years) |  |  |  |  |  | 0.783^*^ |
| <35 | 2 | 853 | 1153 | 0% | 0.19 (0.14-0.25) | <0.001 |
| ≥35 | 3 | 135 | 531 | 0% | 0.21 (0.10-0.43) | <0.001 |
| Quality |  |  |  |  |  | - |
| G/GG | 3 | 1009 | 1611 | 0% | 0.21 (0.16-0.27) | <0.001 |
| G/GP | 3 | 1006 | 506 | 0% | 0.22 (0.16-0.30) | <0.001 |
| P/PP | 1 | 50 | 147 | - | 0.09 (0.02-0.38) | 0.001 |
| Embryo stage |  |  |  |  |  | 0.629^*^ |
| Cleavage | 3 | 59 | 123 | 0% | 0.15 (0.05-0.49) | 0.002 |
| Blastocyst | 5 | 1167 | 2513 | 0% | 0.20 (0.16-0.26) | <0.001 |
| Cycle |  |  |  |  |  | 0.937^*^ |
| Fresh | 6 | 301 | 490 | 0% | 0.20 (0.13-0.31) | <0.001 |
| Frozen | 3 | 1059 | 2336 | 0% | 0.20 (0.16-0.26) | <0.001 |
| Design |  |  |  |  |  | 0.964^*^ |
| RCT | 2 | 37 | 65 | 0% | 0.21 (0.06-0.76) | 0.018 |
| Observational study | 7 | 1323 | 2761 | 0% | 0.20 (0.16-0.25) | <0.001 |
| **Sensitivity analyses** | | | | | |  |
| Adjusted | 3 | 228 | 930 | 0% | 0.30 (0.19-0.48) | <0.001 |
| First cycle | 1 | 908 | 1427 | - | 0.19 (0.14-0.25) | <0.001 |
| eSET | 3 | 233 | 383 | 0% | 0.19 (0.12-0.31) | <0.001 |
| Note:  Abbreviation: CI, confidence interval; DET, double embryo transfer; eSET, elective single embryo transfer; G/GG, a single good quality embryo (GQE) compared with two GQEs; G/GP, a single GQE compared with two embryos of mixed quality (GQE+PQE); OR, odds ratio; P/PP, a single non-top quality embryo (PQE) compared with two PQEs; RCT, randomized controlled trial; SET, single embryo transfer.   ^*^, P for interaction. | | | | | | |
|  |  |  |  |  |  |  |
|  |  |  |  |  |  |  |
|  |  |  |  |  |  |  |
|  |  |  |  |  |  |  |
|  |  |  |  |  |  |  |
|  |  |  |  |  |  |  |
