# Supplementary figures and images for: Comparisons of benefits and risks of single embryo transfer versus double embryo transfer: a systematic review and meta-analysis
Source: Reprod Biol Endocrinol. 2022 Jan 27;20:20. doi: 10.1186/s12958-022-00899-1 (PMC8793185; doi:10.1186/s12958-022-00899-1)

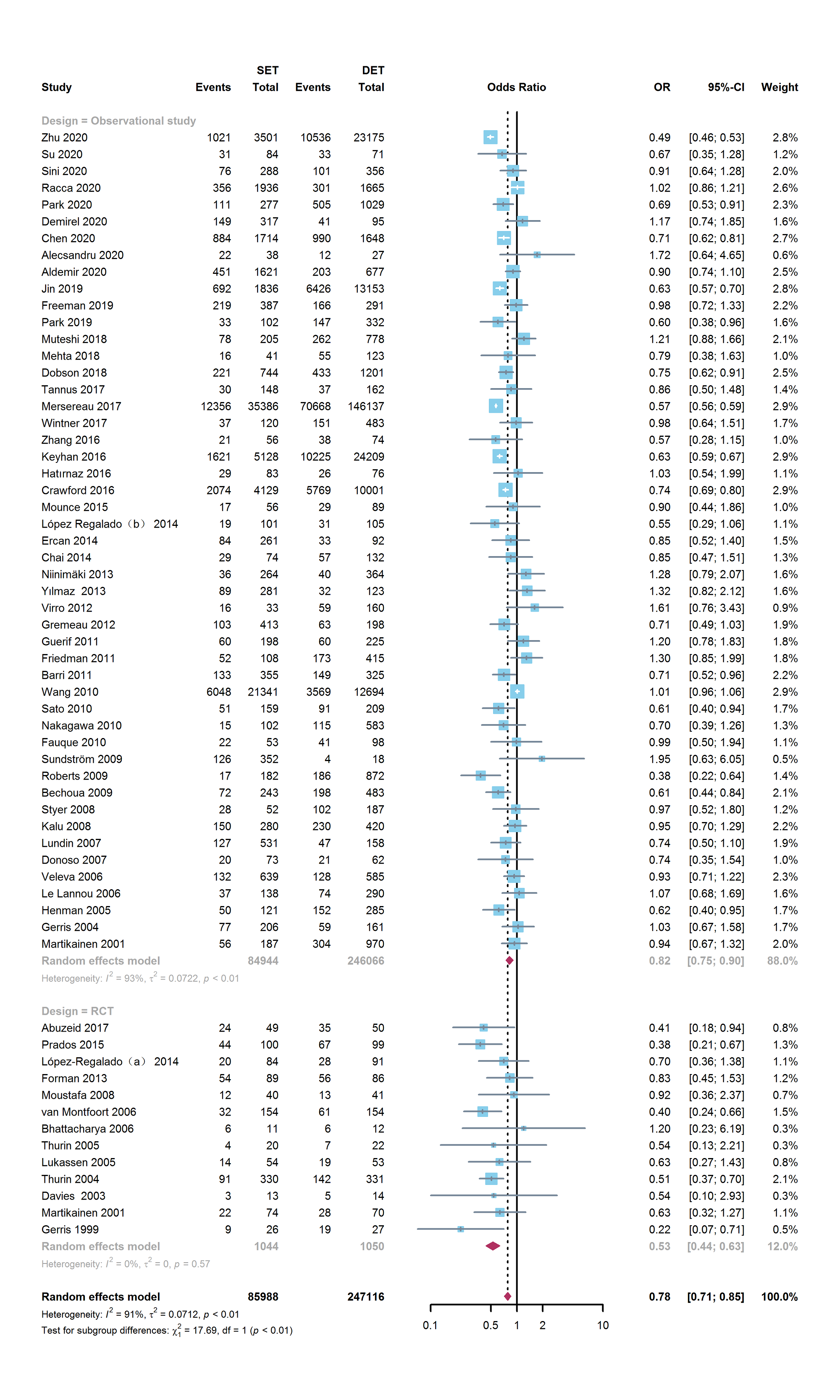

Supplement: Supplementary file 3 — Additional file 3: Figure S1. Forest-plot comparing live birth rate after single embryo transfer (SET) and double embryo transfer (DET) in a single cycle. [file 12958_2022_899_MOESM3_ESM.tif]

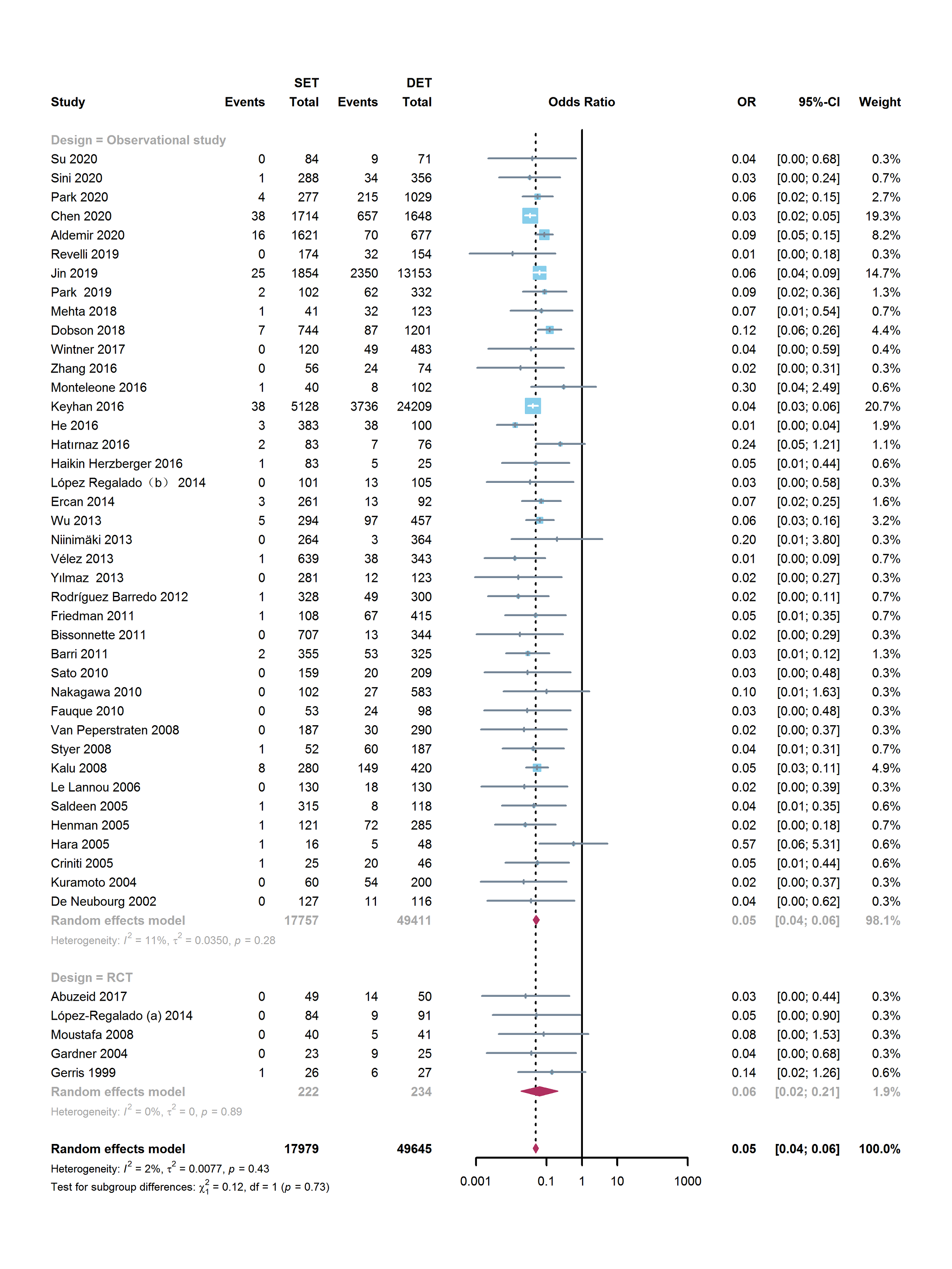

Supplement: Supplementary file 4 — Additional file 4: Figure S2. Forest-plot comparing multiple pregnancy rate after single embryo transfer (SET) and double embryo transfer (DET) in a single cycle. [file 12958_2022_899_MOESM4_ESM.tif]

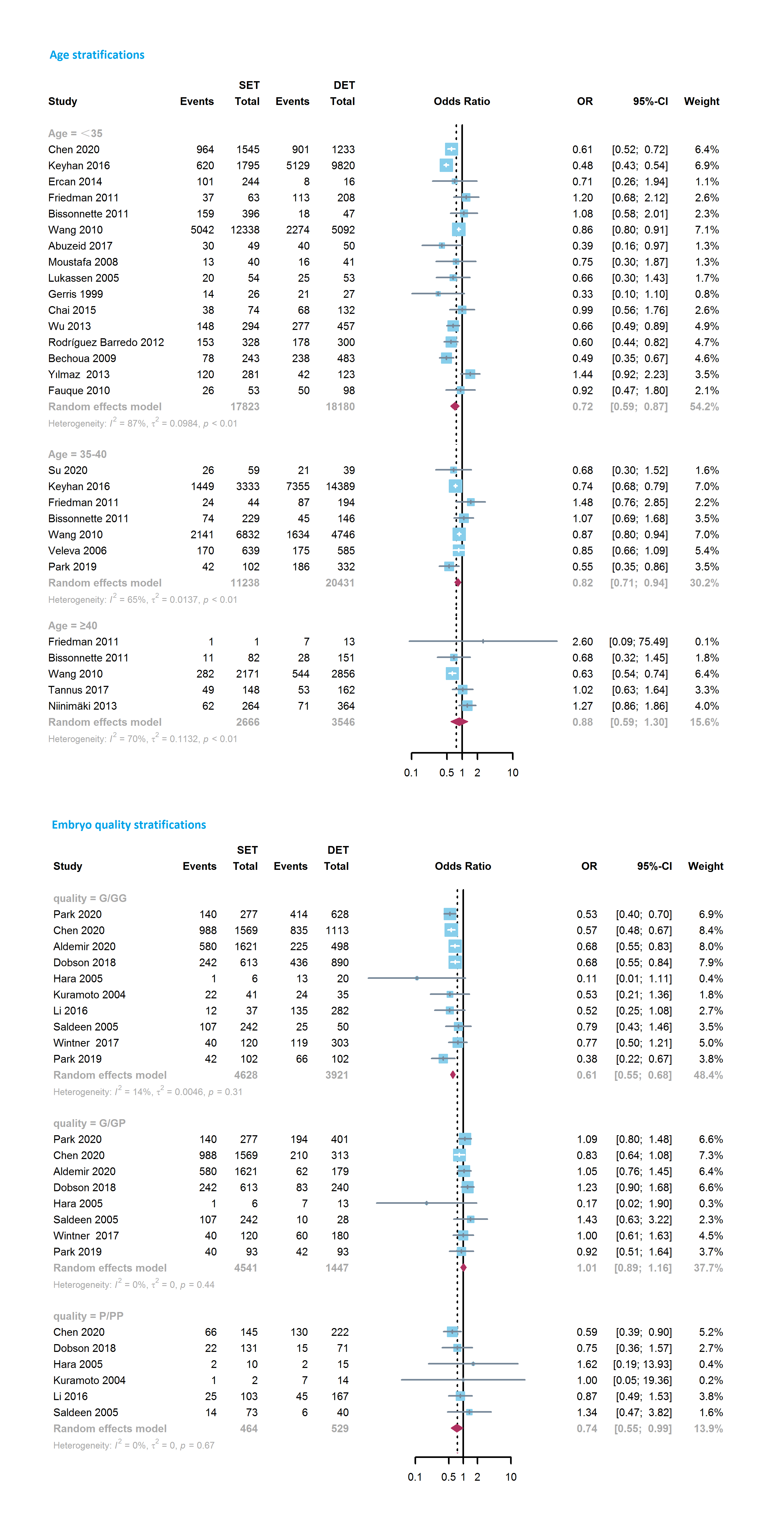

Supplement: Supplementary file 5 — Additional file 5: Figure S3. Forest-plot comparing the clinical pregnancy rate between single embryo transfer (SET) and double embryo transfer (DET) based on maternal age and embryo quality stratification. G/GG, a single good quality embryo (GQE) versus two GQEs; P/PP, a single non-good quality embryo (PQE) versus two PQEs; G/GP, a single GQE versus two embryos of mixed quality (GQE + PQE). [file 12958_2022_899_MOESM5_ESM.tif]
